# Berry-Enriched Diet in Salt-Sensitive Hypertensive Rats: Metabolic Fate of (Poly)Phenols and the Role of Gut Microbiota

**DOI:** 10.3390/nu11112634

**Published:** 2019-11-03

**Authors:** Andreia Gomes, Carole Oudot, Alba Macià, Alexandre Foito, Diogo Carregosa, Derek Stewart, Tom Van de Wiele, David Berry, Maria-José Motilva, Catherine Brenner, Cláudia Nunes dos Santos

**Affiliations:** 1 Instituto de Biologia Experimental e Tecnológica, Apartado 12, 2780-901 Oeiras, Portugal; andreia.gomes@nms.unl.pt (A.G.); diogo.carregosa@nms.unl.pt (D.C.); 2 Instituto de Tecnologia Química e Biológica, Universidade Nova de Lisboa, Av. da República, 2780-157 Oeiras, Portugal; 3 INSERM UMR-S 1180- University Paris-Sud, University Paris Saclay, 5 rue Jean-Baptiste Clément, 92296 Châtenay Malabry, France; oudot.carole@gmail.com (C.O.); catherinebrenner@yahoo.com (C.B.); 4 Food Technology Department, Agrotecnio Center, Escuela Técnica Superior de Ingeniería Agraria, University of Lleida, 25198-Lleida, Spain; albamacia@tecal.udl.cat; 5 Environmental and Biochemical Sciences, James Hutton Institute, Invergowrie, Dundee, Scotland DD2 5DA, UK; alex.foito@hutton.ac.uk (A.F.); derek.Stewart@hutton.ac.uk (D.S.); 6 CEDOC, Chronic Diseases Research Centre, NOVA Medical School|Faculdade de Ciências Médicas, Universidade NOVA de Lisboa, Campo dos Mártires da Pátria, 130, 1169-056 Lisboa, Portugal; 7 Institute of Mechanical Process and Energy Engineering, School of Engineering and Physical Sciences, Heriot Watt University, Edinburgh, Scotland EH14 4AS, UK; 8 Center for Microbial Ecology and Technology (CMET), Ghent University, Coupure Links 653, 9000 Ghent, Belgium; Tom.VandeWiele@ugent.be; 9 Division of Microbial Ecology, Department of Microbiology and Ecosystem Science, Research Network Chemistry Meets Microbiology, University of Vienna, Althanstrasse 14, A-1090 Vienna, Austria; berry@microbial-ecology.net; 10 Instituto de Ciencias de la Vid y del Vino-ICVV, CSIC-Universidad de La Rioja-Gobierno de La Rioja, Finca “La Grajera”, Carretera de Burgos km 6, 26007 Logroño, Spain; motilva@icvv.es

**Keywords:** polyphenols, cardiovascular, dysbiosis, gut metabolism, high salt intake

## Abstract

Diets rich in (poly)phenols are associated with a reduced reduction in the incidence of cardiovascular disorders. While the absorption and metabolism of (poly)phenols has been described, it is not clear how their metabolic fate is affected under pathological conditions. This study evaluated the metabolic fate of berry (poly)phenols in an in vivo model of hypertension as well as the associated microbiota response. Dahl salt-sensitive rats were fed either a low-salt diet (0.26% NaCl) or a high-salt diet (8% NaCl), with or without a berry mixture (blueberries, blackberries, raspberries, Portuguese crowberry and strawberry tree fruit) for 9 weeks. The salt-enriched diet promoted an increase in the urinary excretion of berry (poly)phenol metabolites, while the abundance of these metabolites decreased in faeces, as revealed by UPLC–MS/MS. Moreover, salt and berries modulated gut microbiota composition as demonstrated by 16S rRNA analysis. Some changes in the microbiota composition were associated with the high-salt diet and revealed an expansion of the families *Proteobacteria* and *Erysipelotrichaceae*. However, this effect was mitigated by the dietary supplementation with berries. Alterations in the metabolic fate of (poly)phenols occur in parallel with the modulation of gut microbiota in hypertensive rats. Thus, beneficial effects of (poly)phenols could be related with these interlinked modifications, between metabolites and microbiota environments.

## 1. Introduction

Cardiovascular diseases (CVDs) are the leading cause of death globally, accounting for more than 17 million deaths, meaning that more people die annually from CVDs than from any other cause [1]. CVDs are a group of disorders affecting the heart and blood vessels, and include diseases and/or syndromes such as atherosclerosis and hypertension, ischemic coronary and heart diseases, myocardial infarction, and heart failure [1,2]. Most of these diseases are heterogeneous conditions with diverse aetiologies and comorbidities, generally aggravated by age and risk factors such as family history, gender, tobacco and alcohol consumption, unhealthy diet, physical inactivity, cholesterol, and hypertension. Due to their prevalence and their anticipated increase in the next decade [1], the study of CVDs and the search for better treatments have become an increasing focus of interest for the academic and pharmaceutical sectors.

Cardiovascular health is affected by alterations in human dietary and lifestyle patterns. A high salt intake is associated with an increased risk of hypertension and cardiovascular diseases [3,4,5]. It has been estimated that excess sodium intake was responsible for 1.7 million deaths from cardiovascular causes globally in 2010 [5]. For a long time, the deleterious effects associated with high salt consumption were only related to the effect of salt on blood pressure (BP) [6,7,8]. However, numerous other effects have been reported, such as endothelial dysfunction, stroke, ventricular hypertrophy and fibrosis, arterial and ventricular stiffening, myocardial infarction, arrhythmias, and heart failure [6].

Several epidemiological studies have suggested an association between (poly)phenol-enriched diets (fruits and vegetables) and a lower risk of CVDs [9]. Berries are among the fruits with higher (poly)phenol content and, although limited, there is data supporting their recommendation as an essential part of a diet beneficial to the heart [10].

Through the years, intervention studies have investigated the effects of different berries (i.e., blackcurrants, bilberries, blueberries, chokeberries, cranberries, lingonberries, raspberries, strawberries among others) in healthy human subjects or in subjects with CVD risk factors. Reviewed by [10]. The underlying mechanisms for these beneficial effects are believed to include the upregulation of endothelial nitric oxide synthase (eNOS), decreased oxidative stress, the attenuation of inflammatory gene expression including the reduction of inducible nitric oxide synthase (iNOS) and foam cell formation [10,11]. A significant reduction in CVD mortality, associated with strawberry intake, during a 16 year follow-up period was observed in post-menopausal women. Moreover, for strawberries and blueberries, the significant reduction in the relative risk was associated with at least once per week consumption [12]. Moreover, specific berries, such as bilberry and blackcurrant extracts, chokeberry juice, cranberry extracts, and freeze-dried strawberries were shown to have favourable effects on plasma glucose or lipid profiles in subjects with metabolic risk factors including type 1 or type 2 diabetes mellitus, dyslipidemia, or metabolic syndrome [13,14,15,16]. These studies ranged in duration from 4 to 12 weeks and used conventional berry products or purified anthocyanin extracts, suggesting that both these forms of delivery are effective.

Regarding the effects in blood pressure, trials conducted using different varieties of fresh and processed berry products, showed a significant decrease in systolic blood pressure; in healthy men following cranberry juice supplementation [17]; in subjects with CVD risk factors following mixed berry supplementation [18]; and in postmenopausal women with pre- and stage 1 hypertension following daily blueberry and strawberry consumption for 8 weeks [19,20]. However, the cellular processes and molecular targets of berries (poly)phenols remain to be identified, and therefore there is a need for more studies of berry supplementation as a potential dietary therapy for the management of pre-hypertension or hypertension.

We recently demonstrated the beneficial effects of berry-enriched diet ingestion in a chronic model of hypertension-induced heart failure, the Dahl salt-sensitive (Dahl/SS) rats, and underpin possible molecular targets [21]. The berry mixture provided to the rats prolonged the lifespan, maintained the body weight, ameliorated systolic blood pressure (SPB) and cardiac function and decreased cardiac and renal hypertrophy of rats fed a high-salt diet [21]. The study clearly supports the modulation of cystein and glycin-rich protein 3 (CSRP3) involved in myocyte cytoarchitecture, by the (poly)phenol-rich berry diet as an efficient cardioprotective strategy in hypertension-induced heart failure. Yet, it was not clarified how berry’s (poly)phenols metabolism was affected under these pathological conditions. The elucidation of how they are absorbed, metabolised, and eliminated from the body is crucial to ascertain their in vivo actions.

(Poly)phenol metabolites can be absorbed in the stomach, small intestine, or in the colon [11]. However, their bioavailability in humans is very low. In the upper gastrointestinal tract, the absorption starts with cleavage of the sugar moiety and diffusion of the aglycone to the enterocytes. Before entering systemic circulation, (poly)phenols may undergo phase II metabolism reactions (the formation of glucuronic, sulphate, and/or methyl derivatives). Phenolic compounds that are not absorbed in the small intestine will reach the colon, where they are extensively metabolised by the colonic microbiota into a series of simple phenolic metabolites [11]. These metabolites, being absorbable, may be the ones responsible for the health benefits derived from (poly)phenols-rich food consumption, rather than the parent compounds per se, which are underrepresented in circulation [11,22,23,24]. Conversely, phenolic compounds are described to modulate gut microbiota by the inhibition of pathogenic bacteria and the stimulation of beneficial bacteria. This bidirectional relationship may contribute to host health benefits, although the mechanisms are not fully understood [11,22,25,26].

Until now, only few bacterial species (e.g., *Escherichia coli*, *Bifidobacterium* sp., *Lactobacillus* sp., *Bacteroides* sp., and *Eubacterium* sp.) have been identified as responsible for the catabolism of the phenolic compounds and their pathways [27]. Thus, the effect of dietary (poly)phenols on the modulation of the gut microbiota, including the underlying mechanisms and the actual benefits of such bioactive compounds, remains poorly understood. Moreover it is not clear how the composition of the gut microbiota is modulated not only in healthy individuals, but also under a disease status, thereby providing a link between bioactive metabolites of (poly)phenols and their health benefits [28].

This work aims to address this knowledge gap by providing evidence of the metabolic fate of dietary (poly)phenols, in a rat model of hypertension where we previously described that they developed elevated systolic blood pressure (SPB), cardiac dysfunction and hypertrophy [21], as well as evaluating alterations in the model’s gut microbiota composition by this (poly)phenol rich diet in the context of hypertension. In this work, the metabolic fate of (poly)phenols in an animal model of hypertension (Dahl/SS rats) was evaluated using a quantitative analysis of tissues, liver and heart, and also blood, urine and faeces revealing that a high-salt diet exerts an influence in the excretion of (poly)phenol metabolites. Both dietary salt content and berry consumption induced changes in the gut microbiota, for example berry consumption was associated with an expansion of the phylum Bacteroidetes and a decrease in Firmicutes in both low-salt (LS) and high-salt (HS) diets. Interestingly, Proteobacteria were elevated in the HS diet, but berry consumption led to a reduction in their abundance.

## 2. Experimental Section

### 2.1. Chemicals and Reagents

Standards of catechol, (−)-epicatechin, (+)-catechin, (−)-epigallocatechin, (−)-epigallocatechin-3-*O*-gallate, gallic acid, 4-*O*-methylgallic acid, *p*-hydroxybenzoic acid, protocatechuic acid, 2-(phenyl) acetic acid, 3-(4-hydroxyphenyl)propionic acid, *p*-coumaric acid, ferulic acid, ellagic acid, caffeic aid, gallic acid, protocatechuic acid, dihydromyricetin, eriodictyol, 7-hydroxyflavonol, myricetin, quercetin, kaempferol, cyanidin and morin were purchased from Sigma Aldrich (Dorset, UK) or Extrasynthese Ltd. (Genay, France).

The procyanidin dimer B_2_ [epicatechin-(4β-8)-epicatechin], 2-(2-hydroxyphenyl) acetic acid, 2-(4-hydroxyphenyl) acetic acid and 3-(2,4-dihydroxyphenyl)propionic acid were purchased from Fluka Co. (Buchs, Switzerland). The 4-*O*-methylgallic acid sulphate, catechol sulphate, pyrogallol sulphate, 4-*O*-methylcatechol sulphate, vanillic acid sulphate, and 1-*O*-methylpyrogallol sulphate were chemically synthetised in collaboration with Dr. Rita Ventura as previously published [29]. The reagents formic acid, ethyl acetate, sulphuric acid, hydrochloric acid, sodium hydroxide, petroleum ether, cellulase (EC 3.2.1.4) and hesperidinase (EC 3.2.1.40) from *Aspergillus niger* were purchased from Sigma-Aldrich (St. Louis, MO, USA).

The acetonitrile (HPLC-grade), methanol (HPLC-grade), acetone (HPLC-grade) and glacial acetic acid (99.8%) were of analytical grade (Scharlab, Barcelona, Spain or JT Baker brand (Scientific Chemical Supplies, UK)). Ortho-phosphoric acid 85% was purchased from MontPlet and Esteban S.A. (Barcelona, Spain). L-(+)-ascorbic acid (reagent grade) was provided by Scharlau Chemie (Barcelona, Spain). Ethanol was purchased from Carlo Erba (Laborspirit, Lda. Lisbon, Portugal). Sodium acetate was purchased from Merck (Darmstadt, Germany). Ultrapure water was obtained from a Milli-Q water purification system (Millipore Corp., Bedford, MA, USA).

### 2.2. Preparation and Characterization of Diets

#### 2.2.1. Fruit Puree (Berry Mixture)

The fruit puree consisted of equal parts of five fruits (3.7 kg): southern highbush blueberries (*Vaccinum corymbosum* L. variety Georgia Gem), blackberries (*Rubus* L. subgenus *Rubus Watson* variety Karaka Black) and raspberries (*Rubus idaeus* L. variety Himbo Top) were harvested at the Fataca experimental field in Odemira, Portugal; strawberry tree fruits (*Arbutus unedo* L.) were harvested in the Alentejo region, Portugal and Portuguese crowberries (*Corema album* L.) were harvested in the Comporta region, Portugal. Fruits were blended together using a domestic food processor at room temperature. The resulting puree was freeze-dried and stored at −20 °C until further use. Detailed identification and semi-quantification of (poly)phenols in the fruit purée has been described previously [30]. These have been analysed by HPLC–MS, as it is reported in Section 2.2.5.

#### 2.2.2. Low-Salt and High-Salt diets

Rat powder chow (50 g) was prepared fresh every day by mixing the regular AIN-76a (INRA UPAE, Jouy-en Josas, France) with or without a lyophilised berry mixture: (1) a low-salt diet enriched with 0.26% NaCl (LS); (2) a low-salt diet enriched with 0.26% NaCl and supplemented with 2 g of lyophilised berry mixture (LSB); (3) a hypersaline diet enriched with 8% NaCl (HS); and (4) a hypersaline diet enriched with 8% NaCl, and supplemented with 2 g of lyophilised berry mixture (HSB).

#### 2.2.3. Determination of Proximate Composition

The proximate composition of the feeds (LS, LSB, HS and HSB) was determined based on the standard methods of the Association of Official Analytical Chemists (AOAC) [31]. The water content was determined by drying at 103 ± 2 °C to a constant mass. Ashes content was obtained by drying at 550 ± 50 °C for 6 h. Total nitrogen was obtained by Kjeldahl method [32]: digestion with sulphuric acid at 410 °C for 40 min, followed by distillation with sodium hydroxide and titration with hydrochloric acid. To obtain crude protein, total nitrogen content was multiplied by a conversion factor of 6.25. Crude fat content was determined by extraction with petroleum ether in a Soxhlet apparatus for 60 min, followed by solvent evaporation at 103 ± 2 °C to a constant mass. Crude fibre was determined according to Weende method as described by AOAC [31], by digesting the sample in refluxing 1.25% sulphuric acid (30 min), followed by 1.25% sodium hydroxide (30 min). After digestion, samples were dried at 105 °C for 1 h and heated at 550 °C for 3 h. Carbohydrates were calculated by difference (subtracting water, ashes, protein, fats and fibres).

#### 2.2.4. Enzymatic Hydrolysis of Glycosides

Enzymatic hydrolysis of glycosides was performed according to Pimpão et al. (2013) [30] with slight adjustments. Briefly, phytochemicals were extracted by homogenizing 2.5 g of lyophilised berry mixture, LS, LSB, HS and HSB diets with 7.5 mL of methanol/0.2% acetic acid containing 0.05 mg/mL of morin as internal standard. Samples were vortexed and shaken for 30 min at room temperature in the dark. The mixture was then centrifuged at 12400× *g* for 10 min at room temperature. Supernatants were filtered through a 0.2 μm Teflon (PTFE) filter followed by drying under a nitrogen flow and stored at −80 °C until further use.

Samples for aglycone analysis were reconstituted in water with 1 mM ascorbic acid, and then adjusted to pH 3.8 with 0.2 M acetate buffer and incubated for 16 h at 40 °C with 0.02 U/mL hesperidinase [1 unit (U) corresponds to 333 mg of protein and is defined as the amount required to liberate 1.0 μmol of glucose from hesperidin per minute at pH 3.8 at 40 °C]. The pH was then increased to 5.0 with sodium acetate, and cellulase was added to a final concentration of 20 U/mL (1 U corresponds to 0.885 mg of protein and is defined as the amount required to liberate 1.0 μmol of glucose from cellulose for 1 h, at pH 5.0 at 37 °C). Samples were incubated for 4 h at 37 °C and then extracted three times with ethyl acetate. The ethyl acetate phase (containing phenolic aglycones) was separated and evaporated with a nitrogen flow, then the dried samples were reconstituted with 14 mL of 0.1% (*w*/*v*) formic acid solution in acetonitrile/water (1:1) and filtered through a 0.2 μm PTFE filter prior to HPLC–MS analysis. The procedure was performed in triplicate for each diet.

#### 2.2.5. HPLC–MS Analysis of (Poly)Phenols from Diets

Hydrolysed and non-hydrolysed lyophilised berry mixture and diets were analysed by HPLC–MS. The analysis was conducted on an Accela 600 HPLC system (Thermo Scientific, Bremen, Germany) using a 150 × 2 mm C_18_ Synergi Hydro RP_18_ with 4 µm particle size (Phenomenex, Macclesfield, UK) fitted with a security Guard system containing Aqua 10 m and C_18_ Guard cartridge (2 mm × 4 mm). The elution gradient was achieved with dH_2_O with 0.1% formic acid (solvent A) and acetonitrile with 0.1% formic acid (solvent B). The gradient program used started with 2%B and held for 2 min, increased to achieve 5%B at 5 min, 45%B at 25 min and 100%B at 26 min. The gradient was held at 100% for 3 min, subsequently brought back to 2%B by 30 min, and then held this gradient for 5 min. Analysis was performed on an LTQ Orbitrap XL hybrid mass spectrometer (Thermo Scientific, Bremen, Germany). The electrospray ionization (ESI) settings were as follows: the capillary temperature was 380 °C with sheath gas at 35 psi and auxiliary gas at 15 psi. For the positive polarity, the source voltage was set at 4.50 kV, source current at 110 µA, capillary voltage at 44.00 V and tube lens voltage at 120 V. For negative polarity, the source voltage was set at 3.50 kV, source current at 100 µA, capillary voltage at −44.00 V and tube lens voltage at −100.00 V. The MS analysis was performed using data-dependent Nth order double play analysis comprising a full scan mass range 80–2000 amu, 30,000 resolution data-type profile and data-dependent MS/MS on the top five most intense peaks (signal threshold of 600,000 and 300,000 counts in positive and negative mode, respectively). MS data handing software (Xcalibur Qualbrowser and QuantBrowser, Thermo Electron Corp) was used to search for metabolites and their appropriate *m*/*z* values and perform semiquantitative analysis of target compounds. All peaks were checked for *m*/*z* value, retention time and fragmentation products for identification purposes. Based on an initial qualitative analysis of the dataset a targeted list of compounds was selected for semi-quantification using total areas of selected ion chromatograms. Principal Component Analysis (PCA) was used to summarise broad scale variation among hydrolysed and non-hydrolysed samples in both positive and negative modes. Analysis of variance was performed to compare high salt vs. low salt in berry diets individually for hydrolysed and non-hydrolysed datasets as well as ESI positive and ESI negative datasets. Statistical analysis was performed on GenStat for Windows version 15.3.0.9425 (VSN International Ltd., Hemel Hempstead, UK).

### 2.3. Animals

Male Dahl/SS rats (obtained from Charles River) weighing 169 ± 7 g were used in the experiments. Forty animals were housed two per cage in a temperature (22 ± 1 °C) and humidity (55 ± 10%) controlled room with a 12 h light/dark cycle (lights on at 08:00 h). The experiments were performed during the light period. Food and water were available *ad libitum* in both the home and metabolic cages.

After 1 week of acclimatization, rats were divided into 4 groups for ten weeks. Rats were fed a AIN-76a powder chow (INRA UPAE, Jouy-en Josas, France) with or without berry mixture and with two levels of salt content, low or high (LS, LSB, HS, HSB).

For the collection of urine and faeces, the rats were allocated one per metabolic cage for 4 days, with a 24 h adaptation period before urine and faeces collection in the 4th and 9th week. After collection, urine was centrifuged at 3000× *g* for 5 min at 7 °C. A total of 500 μL of urine was acidified with 100 μL of Milli-Q water/formic acid (50/50, *v*/*v*). Samples were stored at −20 °C until further analysis. Faeces were frozen in liquid N_2_ immediately after collection and stored at −80 °C until further analysis.

At the end of the experiment, animals were sacrificed by decapitation without any anaesthetic previously prescribed. Blood was collected and centrifuged at 2000× *g* for 10 min to obtain plasma. Fifty μL of 10 mM ascorbic acid solution prepared in 50% Milli-Q water/formic acid (*v*/*v*) was added to 1 mL of plasma to limit oxidation and acidify the solution. Samples were stored at −20 °C until further analysis.

Different tissues (liver and heart) were excised from the rats, frozen in liquid N_2_ immediately after collection followed by storage at −80 °C until further use.

The Ethical Committee of the University of Paris-Sud, France approved all animal research protocols (N°APAFIS#2152-2015092912128144 v3). All animal care and experimental procedures were performed according to the European Community guiding principles in the care and use of animals (Directive 2010/63/EU of the European Parliament) and authorizations to perform animal experiments according to this directive were obtained from the French Ministry of Agriculture, Fisheries and Food (No. D-92-283, 13 December 2012) All studies involving rats are reported in accordance with the Animal Research: Reporting of *In Vivo* Experiments (ARRIVE) guidelines for reporting experiments involving animals.

### 2.4. Plasma, Urine, Faeces and Tissues Treatment

In order to clean up the biological matrix and pre-concentrate the phenolic compounds, a pre-treatment with microelution solid-phase extraction (μSPE) was used for plasma and urine [33,34,35]. Regarding faeces and tissues (liver and heart), they were sequentially pre-treated by a combination of liquid–solid extraction (LSE) and SPE [36]. The methodologies were followed according to previous publications [37].

#### 2.4.1. Plasma Samples

For plasma, 350 μL of the sample was combined with 350 μL of phosphoric acid (4%) and centrifuged at 8784× *g* for 10 min (Hettich Universal 320-R, Tuttlingen, Germany). The collected supernatant was pre-treated by µSPE as described in previous studies [33,34]. Briefly, the micro-cartridges OASIS HLB µElution Plates 30 mm (2 mg) (Waters, Milford, MA, USA) were conditioned sequentially by using 250 µL of methanol and 250 µL of 0.2% of acetic acid, and then the sample was loaded onto the plate. The loaded plates were washed with 200 µL of Milli-Q water and 200 µL of 0.2% acetic acid. Then, the retained phenolics were eluted with 2 × 50 µL of acetone/Milli-Q water/acetic acid solution (70/29.5/0.5, *v*/*v*/*v*). The eluted solution was directly injected into the chromatographic system.

#### 2.4.2. Urine Samples

For urine, 100 μL of the sample was mixed with 50 µL of catechol (20 mg/L) as internal standard (it was prepared with phosphoric acid at 4%), followed by centrifugation at 8784× *g* for 10 min. The supernatants were collected, and an aliquot of the extract was pre-treated by µSPE. The micro-cartridges were conditioned sequentially with 250 µL of methanol and 250 µL of 0.2% of acetic acid. Then, the retained phenolic compounds were eluted with 2 × 50 µL of acetone/Milli-Q water/acetic acid solution (70/29.5/0.5, *v*/*v*/*v*) and directly analysed into the chromatographic system.

#### 2.4.3. Faecal Samples

For faecal samples, a liquid–solid extraction was carried out by adding 1 mL of methanol/Milli Q water/HCl (79.9/20/0.1, *v*/*v*/*v*) to 100 mg of lyophilised faeces. The samples were vortexed for 10 min at room temperature. The samples were then centrifuged twice (10 min, 8784× *g*, at room temperature) and the resultant supernatant was filtered with a 0.22 μm nylon syringe filter and transferred into chromatographic vials for analysis.

#### 2.4.4. Tissues Samples (Liver and Heart)

Briefly, for the analysis of the tissues, 60 mg of freeze-dried liver and 50 mg of heart were mixed with 50 µL of ascorbic acid (1%), and 150 µL of phosphoric acid (4%). Each sample was extracted four times with 400 µL of extraction solution (Milli-Q water/methanol/phosphoric acid 4%; 94/4.5/1.5, *v*/*v*/*v*). The samples were then sonicated (S-150D Digital SonifierR Cell Disruptor, Branson, Ultrasonidos S.A.E., Barcelona, Spain) for 30 s, maintaining the sample in an ice water bath, and then centrifuged for 15 min at 8784× *g* at 20 °C.

Aliquots of the extracts were pre-treated by SPE (OASIS HLB 3 cc (60 mg) from Waters, Milford, MA, USA) in order to clean up and pre-concentrate the sample before chromatographic analysis. Briefly, the cartridges were conditioned sequentially by using 5 mL of methanol and 5 mL of 0.2% acetic acid. Then, 1.4 mL of the tissue extracts was loaded, and the loaded cartridges were washed with 3 mL of Milli-Q water and 3 mL of 0.2% of acetic acid. Finally, the elution of the retained phenolic compounds was performed with 4 mL of acetone/Milli-Q water/acetic acid solution (70/29.5/0.5, *v*/*v*/*v*). Then, the samples were dried under a nitrogen stream. Phenolic compounds were dissolved with 100 μL of the extraction solution, acetone/Milli-Q water/acetic acid solution (70/29.5/0.5, *v*/*v*/*v*), and analysed into the chromatographic system as previously described [34,38].

### 2.5. Analysis of Berries Metabolites by UPLC–ESI-MS/MS

The analysis of berry metabolites in the plasma, urine, faeces and tissues was performed using a UPLC coupled to tandem MS (MS/MS). The chromatographic system is an AcQuity Ultra-Performance^TM^ liquid chromatography and tandem MS from Waters (Milford MA, USA), as reported in a previous study [36]. The column was an Acquity UPLC^TM^ BEH C_18_ (100 × 2.1 mm i.d., 1.7 µm), also from Waters (Milford MA, USA). Two gradients with different mobile phases were used—one for the analysis of anthocyanin metabolites and a second one for the analysis of the remaining phenolic compound metabolites. For the analysis of anthocyanin metabolites, the mobile phase was 10% acetic acid as solvent A, and acetonitrile as solvent B. The flow rate and gradient elution is the reported in our previous study [34]. Briefly, the flow rate was 0.4 mL/min, and the gradient elution was as follows: 0–10 min, 5%–35%B; 10–10.10 min, 35%–80%B; 10.10–11 min, 80%B isocratic; 11–11.10 min, 80%–5%B; 11.10–12.50 min, 5% B isocratic. Regarding the analysis of the rest of the phenolic metabolites, the mobile phase was 0.2% acetic acid as solvent A and acetonitrile as solvent B, and the flow rate was 0.4 mL/min. The gradient elution is as follows: 0–5 min, 5%–10 B%; 5–10 min, 10%–12.4%B; 10–18 min, 12.4%–28%B; 18–23 min, 28%–100%B; 23–25.5 min, 100%B; 25.5–27 min, 100%–5%B; and 27–30 min, 5%B. The injection volume was 2.5 µL.

The AcQuity UPLC^TM^ system was coupled to a PDA detector, and a triple quadrupole detector (TQD^TM^) mass spectrometer (Waters). The software used was MassLynx 4.1. Ionization was performed by electrospray (ESI) in the positive mode for the analysis of anthocyanins, and in negative mode for the remaining phenolic compounds. The ionization source parameters were the reported in a previous study [33,34,35]. The phenolic metabolites derived from berry intake were quantified by using the selected reaction monitoring (SRM) mode. Two transitions were acquired and the most sensitive was used for quantification and the second one for identification purposes. Supplementary Table S1 information shows these SRM transitions used for quantification and identification as well as their cone voltage and collision energy.

### 2.6. 16S rRNA Gene Sequencing Analysis of Gut Microbiota

DNA from primary stool samples were extracted as previously described by Frémont et al. [39]. A normalised quantity of 50 ng of extracted DNA (measured by the Quant-iT PicoGreen dsDNA Assay Kit from Thermo Fisher (Merelbeke, Belgium)) was used for each sample. The 16S rRNA genes were amplified using the S-D-Bact-0341-b-S-17/S-D-Bact-0785-a-A-21 primer pair [40] targeting the amplification of the V3 and V4 hypervariable 16S rRNA gene regions. The 16S Metagenomic Sequencing Library Preparation protocol from Illumina (Part # 15044223 Rev. B) was followed, including the quality controls. The sequencing was carried out on Miseq sequencing machine (Illumina, San Diego, California, USA) with a read length of 2 × 300 nt.

The diversity metrics were calculated using the vegan package in R. Operational taxonomic unit (OTU) tables were subsampled to slightly below the smallest library (30,000 reads) for alpha and beta diversity comparisons. For alpha diversity, OTU richness and Shannon diversity indices were calculated. For beta diversity, Bray–Curtis distance was used for Principal Coordinate Analysis and the clustering of samples was evaluated using analysis of similarity (ANOSIM). Statistical significance of factors shaping the microbiota was determined using permutational multivariate analysis of variance (perMANOVA). Identification of differentially abundant taxa was performed with analysis of variance (ANOVA). P-values were corrected for multiple comparisons using the false discovery rate method.

### 2.7. Short Chain Fatty Acids (SCFAs) Analysis

SCFAs (C2–C8 fatty acids, including isoforms C4–C6) were analysed in faeces, and performed according to Andersen et al. [41]. Briefly, overnight lyophilization was carried out while protecting samples from light. Afterwards, SCFA extraction was carried out with diethyl ether and quantified by gas chromatography (GC-2014, Shimadzu^®^, ’s-Hertogenbosch, The Netherlands) using a DB-FFAP 123-3232 column (30 m × 0.32 mm × 0.25 µm; Agilent, Machelen, Belgium) and a flame ionization detector (FID). Liquid samples were conditioned with sulphuric acid, sodium chloride and 2-methyl hexanoic acid as internal standard for quantification of further extraction with diethyl ether. The prepared sample (1 µL) was injected at 280 °C, with a split ratio of 60:1 and a purge flow of 3 mL/min. The oven temperature gradient increased by 6 °C/min from 110 °C to 158 °C and by 8 °C/min from 158 °C to 175 °C, where it was kept for 1 min using nitrogen as carrier gas at a flow rate of 2.49 mL/min. FID was carried out at 220 °C.

### 2.8. Statistical Analysis

Results are expressed as the mean concentrations (nmol in urine and nmol/g fresh material in faeces) ± Standard Error of Means (SEM). The non-parametric Kruskal–Wallis and Dunn’s tests were applied to analyse significant differences (*p* < 0.05) between groups. The Kruskal–Wallis test was applied to determine whether any significant difference between the treatments were detected. Dunn’s test was subsequently used to compare all the significantly different pairs of the treatments. The Prism 7 for Mac OS X, version 7.0a was used.

## 3. Results

### 3.1. Nutritional Composition of the Diets and Berry Mixture

The proximate compositions of the diets, as well as the berry mixture were determined (Supplementary Tables S2 and S3). The analysis revealed that the dominant components for the LS, LSB, HS and HSB diets are carbohydrates, water and proteins with no statistical differences between samples. The addition of the berry mixture does not alter the nutritional value of the diets.

### 3.2. (Poly)Phenols Content in Diets

Food matrices can interact with (poly)phenols altering their bioavailability. To ensure that the differences observed in polyphenol metabolism of the animals, are not related with food matrix effects, we assessed whether the high-salt content in the diets had an influence in the (poly)phenol content extracted from the berries or on the release of (poly)phenols from the food matrix after digestion.

The analysis of intact, non-hydrolysed LS, LSB, HS and HSB diet extracts by HPLC–MS revealed a great complexity and diversity of compounds particularly in the diets supplemented with berries (Supplementary Tables S4 and S5, and supplementary Figure S1).

To further analyse the impact of salt in the (poly)phenol composition derived from berries, and to maximise the analysis of the differences observed among the samples, the HPLC–MS data was processed by means of Principal Component Analysis (PCA, Supplementary Figure S2). All the diets clustered according to the presence or absence of berries in the diet (Supplementary Figure S2A, PC 1, 92.67%). LS and HS diet samples without berries are clustered together (Supplementary Figure S2A), which demonstrate that the presence of berries in the diet clearly is the main determinant that distinguish the diets. No effect of the salt is evidenced in (poly)phenol composition. Analysis of variance revealed that there were no significant differences (*p* < 0.05) between non-hydrolysed berry diets containing low salt and non-hydrolysed berry diets containing high-salt diets in both ESI positive and negative mode datasets (Supplementary Tables S4 and S5).

The content of anthocyanins in our berry mixture was evaluated since it is described as the major (poly)phenol class represented in the mixture (Supplementary Table S6). Cyanidin glucoside (1053 ± 87.90 mg/kg) was the main anthocyanin quantified in the extract obtained from the berry mixture, followed by delphinidin glucoside (915 ± 64.40 mg/kg), petunidin glucoside (913 ± 68.10 mg/kg) and malvidin glucoside (859 ± 63.10 mg/kg). The less abundant anthocyanins were the ones in the acetylglucoside form. Overall, in each 2 g of lyophilised berry mixture added daily to the rat’s diet, 9.47 mg in total of anthocyanins were incorporated. The main aglycones of the remaining (poly)phenols in the berry mixture were also estimated after enzymatic hydrolysis (Supplementary Table S7), being phenolic acids, the major components followed by flavonols and flavanols.

### 3.3. Analysis of Aglycones after Enzyme Hydrolysis of Glycosides

In order to understand whether the presence of the salt affects the release of the aglycones from the food matrix, and in a certain way, to predict possible effects that occurred during the gastro-intestinal digestion of the Dahl/SS rats, we decided to analyse diets after enzymatic digestion. Extracts from the diets (LS, LSB, HS and HSB) and the berry mixture were incubated with enzymes from *Aspergillus niger*: hesperidinase containing *α*-l-rhamnosidase and *β*-d-glucosidase activities and cellulase containing endo-1,4-*β*-d-glycosidic activity. We used the same strategy as for the non-hydrolysed samples, samples were analysed by HPLC–MS, and data was processed by means of a PCA. Supplementary Figure S2B shows, again, that the presence of berries in the diets is responsible for the differences between the samples (PC 1 88.28%, clusters) and that salt does not affect the release of the aglycones. ANOVA showed that for the positive mode (Supplementary Table S8), there is only one metabolite, out of 144, that is significantly different between LSB and HSB (Unknown 12 C_17_H_19_NO_9_). Regarding the negative mode (Supplementary Table S9), only two metabolites (out of 96) appear to be significantly different between HSB and LSB, gallic acid isomer 1 and syringetin isomer 1, indicating that the presence of salt does not affect significantly the release of the phenolic aglycones. Representative chromatograms of the positive and negative mode of the HPLC–MS analysis are presented in the supplementary Figure S3.

### 3.4. Beneficial Effects of Berries against Chronic Salt Consumption

Recently, our group demonstrated the beneficial effects of the berry-enriched diet used in this trial in the Dahl/SS rats [21]. The goal of this preceding publication was to characterise how this berry mixture protected Dahl salt-sensitive rats from salt-induced cardiovascular damage [21]. No animals from LS and LSB groups died during the trial while in the HS group, mortality reached 40% with rats dying predominantly from stroke. A positive effect of the berry-enriched diet on survival became statistically significant from 6 weeks (*p* < 0.05) until the end of the study, leading to 30% lower animal death in the group with the HS diet supplemented with berries (*p* < 0.05). During the study, no significant differences either in survival rate or body weight gain were observed between LS and LSB rats (Supplementary Figure S4A). In contrast, we observed a significant loss of body weight in the HS rats from 7 weeks (Supplementary Figure S4A). This weight loss was significantly prevented by berry consumption until the end of the trial (Supplementary Figure S4A). Systolic blood pressure (SBP) in HS rats was significantly higher compared to LS animals at 3, 6 and 9 weeks, confirming the deleterious effect of salt on SBP and validating the experimental hypertension model (Supplementary Figure S4B). The SBP of LSB rats tended to be higher at 6 weeks, but after 9 weeks, no differences were found in comparison to LS rats. Interestingly, berries alleviated the development of hypertension induced by HS diet after 3 and 6 weeks. However, SBP of HS rats decreased at 9 weeks and thereby, reached the level of HSB rats (Supplementary Figure S4B). Taken all together, a HS diet promotes animal death, decreases body weight and increases hypertension, while the consumption of berries reverted these deleterious events. Thus, our berry-enriched diet showed cardiovascular protective properties.

### 3.5. Metabolic Fate of (Poly)Phenols

We assessed the metabolic fate of (poly)phenols after the chronic ingestion of the berry-enriched diet for 9 weeks and evaluated the persistence of these metabolites in the organism. Moreover, we aimed to understand whether the metabolic fate of (poly)phenols was affected by the high blood pressure induced by the salt enriched diet. The experiment was not designed as a multi-component pharmacokinetic study, and rats were fasting overnight, approximately for 16 h. With this approach, we were not able to identify any (poly)phenol metabolites in heart, liver, and plasma, indicating that they do not persist in these organs and fluids after 16 h (data not shown).

Food intake was monitored, and no differences were observed between animals with the different diets. The animal’s food intake was between 77% and 86% of the food supplied in the 4th week, with a reduction to 44%–66% at 9 weeks, but again with no significant differences within the groups (data not shown). Regarding water intake, there was a significant increase in rats consuming a HS diet with or without berry supplementation. The water intake in these animals was approximately 2.5 times higher at week 4 and approximately 3 times higher at week 9 of the trial (data not shown) when compared with rats fed a low-salt diet.

#### 3.5.1. Urinary (Poly)Phenols Excretion

A total of 44 urinary (poly)phenol metabolites were identified and quantified by UPLC–MS/MS (Table 1 and Table 2). Table 1 shows the concentration data of 22 metabolites that are present in all rats independently of the diet at 4 and 9 weeks of trial and, therefore, their presence is not specific due to the berry supplementation. However, the levels of most of the metabolites are affected by the supplementation with berries.

Hippuric acid, as expected, was the most abundant phenolic acid in urine samples in the 4 groups and was significantly higher in rats that consumed a berry enriched diet (*p* < 0.001) (Table 1). Interestingly, 4-methyl catechol glucuronide and pyrogallol sulphate were excreted at 4 weeks, but do not appear in urine at the end of the trial (9 weeks). In fact, the major alterations due to the introduction of the salt in the diet were observed at 4 weeks. Further, 3-(hydroxyphenyl) propionic acid sulphate and 3-(4-hydroxyphenyl) propionic acid were present in high levels in rats that ingested the berry mixture during the trial. Interestingly, at 4 weeks, the rats consuming the HSB diet excreted even more of both compounds. Conversely, pyrogallol sulphate, a minor compound present in urine of LS and LSB rats at week 4, exhibited much higher excretion in hypertensive animals where salt is introduced in the diet. In fact, there is a trend for increasing excretion of most of the metabolites (54%) after the 4th week of the introduction of the salt in the diet. However, at week 9, this increase was only observed for 22% of the metabolites. The exceptions were ferulic acid and dihydroferulic acid derivatives, which exhibited a reduced excretion after introduction of the salt.

Of the 44 metabolites identified, half were only excreted in urine of rats with LSB and HSB diet (Table 2). Gallic acid, gallic acid glucuronide, vanillic acid glucuronide, syringic acid, syringic acid sulphate and 5-(dihydroxyphenyl)-γ-valerolactone glucuronide metabolites (Table 2) were only identified in the urine of LSB rats and were absent in that of HSB rats which means that the presence of high amounts of salt in the diet affects their excretion.

Coumaric acid glucuronide was the only metabolite to be exclusively excreted in urine of HSB rats at 4 (1.8 ± 1.0 nmol) and 9 (5.8 ± 1.5 nmol) weeks of diet (Table 2).

For almost half (45%) of the metabolites detected in LSB rats, there was a trend of higher excretion at the end of the trial compared to week 4. This was in contrast to the observations in HSB animals, where only 15% of the metabolites were more excreted at the end of the trial. This reflects an association between the presence of the salt in the diet with the development of any impairment associated to the hypertension.

**Figure 1:**
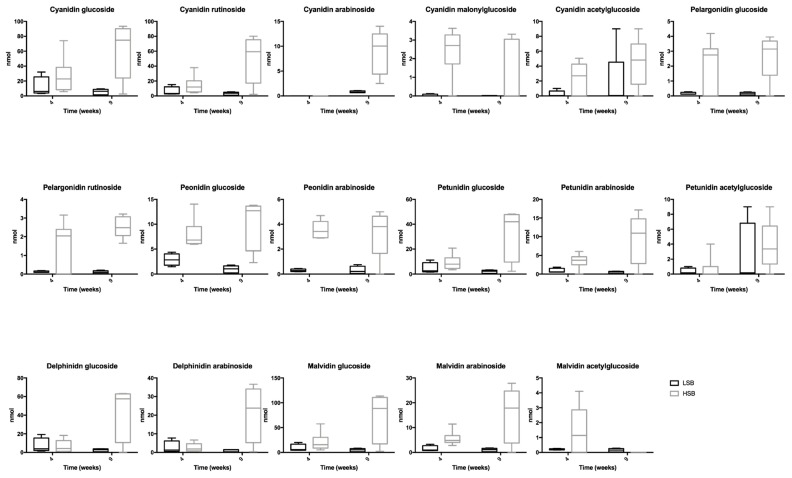
Anthocyanins identified exclusively in urine of rats fed a LSB and HSB diet at 4 and 9 weeks of trial. Results are represented (nmol) as the mean ± SEM of values at 4 and 9 weeks of diet for six rats in the LSB group and 12 rats in the HSB group.

Unsurprisingly, anthocyanins were only identified and quantified in the urine samples of the animals with diets supplemented with berries (Figure 1). For the most abundant anthocyanins (cyanidin glucoside, delphinidin glucoside, petunidin glucoside and malvidin glucoside), there was no detectable difference in the excretion in the hypertensive animals due to the presence of salt in the diet. As previously mentioned, cyanidin glucoside was the main anthocyanin quantified in the extract obtained from the berry mixture and was also the main anthocyanin to be excreted in the urine of LSB and HSB rats.

#### 3.5.2. Faecal (Poly)Phenol Excretion

The analysis of faecal (poly)phenol metabolites revealed a total of 30 metabolites which were identified and quantified by UPLC–MS/MS (Table 3 and Table 4). Further, 2-(Phenyl) acetic acid, 2-(hydroxyphenyl) acetic acid and hippuric acid were the most abundant metabolites found in faeces samples from LS and LSB rats at weeks 4 and 9 of the diet. Interestingly, vanillic acid, vanillic acid sulphate, gallic acid sulphate and caffeic acid were only identified in LS and LSB rats in the 4th week of trial.

Table 4 shows the metabolites that were only excreted by rats that consumed a diet supplemented with berries. Protocatechuic acid was only identified at week 4 in the faeces of rats with LSB and HSB diets, whereas 3-(dihydroxyphenyl)propionic acid was only identified in LSB samples at the end of the trial. Further, 4-*O*-methylgallic acid was another metabolite exclusively excreted in LSB samples. However, it was present at both weeks 4 and 9 of the experiment.

In the animals exposed to high salt in their diets, there was a reduction in the levels of(poly)phenol metabolites excreted. This trend was observed in rats with a HS diet at weeks 4 and 9 of the trial. Moreover, the rate of excretion, independently of the presence of salt, was smaller at week 9 compared to week 4.

**Figure 2:**
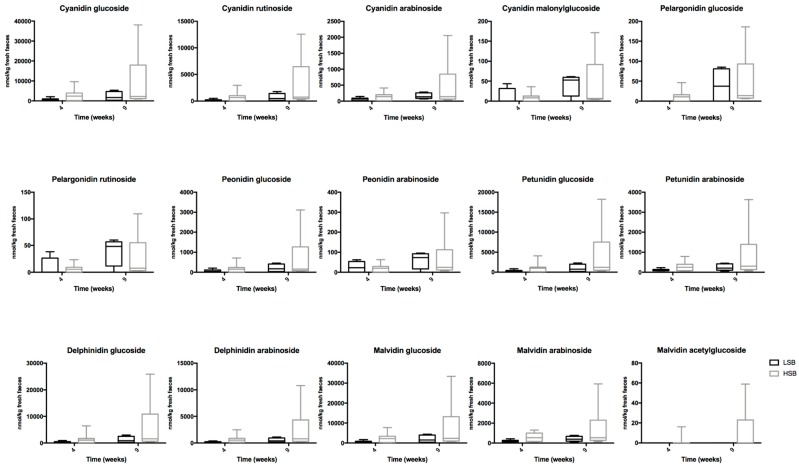
Anthocyanins identified exclusively in faeces of rats fed a LSB and HSB diet at 4 and 9 weeks of trial. Results are represented (nmol/kg fresh faeces) as the mean ± SEM of values at 4 and 9 weeks of diet for six rats in the LSB group and 12 rats in the HSB group.

A total of 15 anthocyanins (Figure 2) were identified and quantified rats with diets supplemented with berries, and in much higher amounts than those found in urine samples. Cyanidin glucoside, cyanidin rutinoside, delphinidin glucoside, delphinidin arabinoside and malvidin glucoside were the major anthocyanins excreted, but there were no detectable differences in the excretion between the control animals and the ones with salt-induced hypertension.

**Figure 3:**
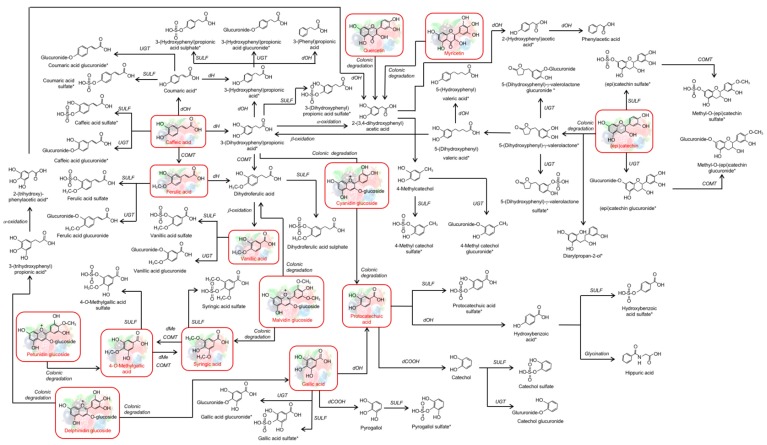
Proposed metabolic pathways of (poly)phenols after ingestion of a berry-enriched diet for 9 weeks. The parent compounds present in the berry mixture are in red box consumed by the rats and the metabolites in black were quantified in urine and faeces. For some compounds it is possible to have more than one isomer in samples. However, is not possible to distinguish them by the MS/MS, we indicated with * the compounds that have other isomer possibilities. dOH—dehydroxylation, dCOOH—decarboxylation, dMe- demethylation, COMT—catechol-*O*-methyl transferase.

Although some of the metabolites detected could be derived from direct absorption and conjugation of (poly)phenols present in the berry mixture, most of those present in urine and faecal samples are derived from gut microbial metabolism. Based on these results, a putative metabolic pathway of berry (poly)phenols after ingestion of a berry-enriched diet for 9 weeks and based in the metabolites excreted was developed (Figure 3).

### 3.6. Salt and Berry Consumption Alter Gut Microbiota Composition

**Figure 4:**
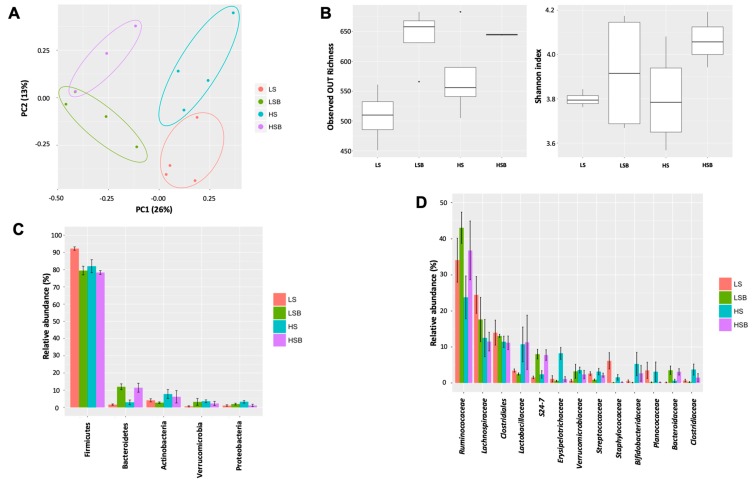
High-salt diet alters gut microbiota composition and function. (**A**) Principal Coordinate Analysis (PCoA) of faecal microbiota composition at 9 weeks exposed to the different diets; (**B**) Alpha diversity analysis (OTU richness and Shannon diversity index); Relative abundance of faecal bacterial at the (**C**) phylum and (**D**) family level. N = four rats per group.

Besides assessing the metabolic fate of (poly)phenols after a chronic ingestion of berries, it is also important to evaluate how this supplementation affects the composition of gut microbiota, which ultimately affects the (poly)phenol metabolism. To investigate how the composition of gut microbiota is affected by the development of a hypertensive phenotype associated with the consumption of high-salt diets and also supplemented with berries, we analysed the faecal microbiota composition and diversity of rats fed with LS, LSB, HS and HSB diets for 9 weeks. The gut microbiota composition was altered by both the hypertensive scenario induced by the salt content of the diet (perMANOVA, *p* = 0.01) as well as the consumption of berries (perMANOVA, *p* = 0.004). Ordination of samples using Principal Coordinate Analysis (PCoA, Bray–Curtis distances) revealed clustering of samples according to diet (ANOSIM, *p* = 0.001) (Figure 4A). The results showed that samples clustered along the first principal coordinate according to the consumption of berries (ANOSIM, *p* = 0.001) and along the second principal coordinate according to the dietary salt intake (ANOSIM, *p* = 0.029). This indicates that both salt and the consumption of berries led to marked and reproducible shifts in the composition of the gut microbiota (Figure 4A).

Alpha diversity analysis revealed that the operational taxonomic unit (OTU) richness (Figure 4B, left panel) was affected by feed regime (ANOVA, *p* = 0.0152), with the consumption of berries increasing microbiota richness (*p* = 0.02). Shannon diversity index (Figure 4B, right panel), which incorporates both richness and evenness of the abundance of OTUs, showed a trend towards increasing diversity with the consumption of berries. However, this is not statistically significant. This indicates that berry consumption promotes the expansion of additional bacterial species (OTUs).

At the phylogenetic level, the consumption of berries led to an expansion of the phylum Bacteroidetes and a decrease in Firmicutes when supplemented in both LS and HS diets (*p* = 0.0004 and *p* = 0.022, respectively) (Figure 4C). Interestingly, Proteobacteria were elevated in the HS diet, but the consumption of berries led to a reduction in their abundance (*p* = 0.033).

In order to better understand the impact of salt-induced hypertension and berry supplementation on the microbial community, we studied the correlation between the exposure to the diet and the taxonomic composition at the family level (Figure 4D). The Bacteroidetes family’s *Bacteroidaceae* and S24-7 were elevated due to the consumption of berries (*p* = 0.029 and *p* = 0.003, respectively). A sharp increase in *Ruminococcaceae* when berries are supplemented in both LS and HS diets was observed (Figure 4D). Also, the family *Erysipelotrichaceae* was increased when the animals were fed a high-salt diet, but the consumption of berries led to a reduction in this group (*p* = 0.01) (Figure 4D).

### 3.7. Salt and Berries Affect SCFA in Faeces

SCFAs are the end products of microbial fermentation and perform diverse functional roles that affect the host’s physiology, and have been linked to blood pressure regulation [42,43,44,45,46]. To examine whether the composition of the rat SCFA was altered as a result of the differential diet, faecal SCFAs were quantitated by gas chromatography.

**Figure 5:**
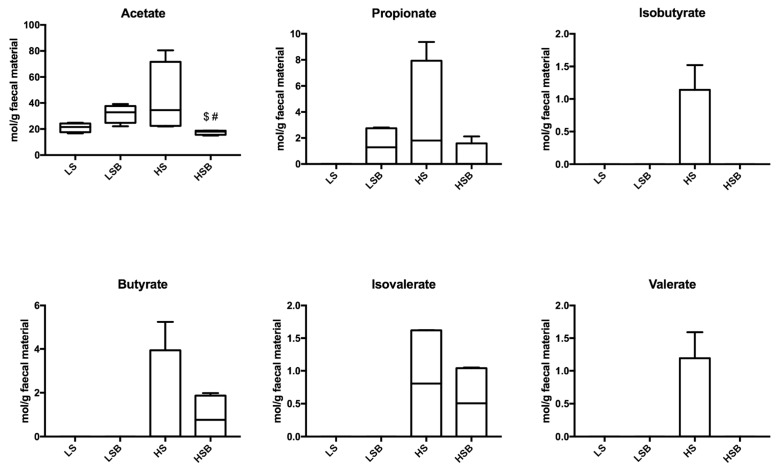
Short Chain Fatty Acids quantification in faeces at the end of the trial. Results are represented (mol/g faecal material) as the mean ± SEM of values at 9 weeks of diet for four rats per group. $ *p* < 0.05 vs. LSB; # *p* < 0.05 vs. HS.

The analysis of the main SCFAs generated by gut microbial fermentation (butyric, propionic, and branched acids) did not show significant changes with any of the interventions (Figure 5). However, acetate was elevated in the faeces of HS rats (42.9 ± 13.6 mol/g faeces), and the introduction of berries in the diet (HSB) promoted a significant decrease in acetate (17.5 ± 0.9 mol/g faeces) production and excretion in faeces.

## 4. Discussion

The deleterious effect of a high-salt diet on cardiovascular health is driven by arterial hypertension and is associated with increased morbidity and mortality [5,47]. Numerous epidemiological studies have shown that a high intake of fruit and vegetables, which are rich sources of (poly)phenols, is associated with a lower incidence of cardiovascular mortality [9] and reduced blood pressure [48,49]. However, is not known how salt or the salt induced pathology can affect the absorption and metabolism of (poly)phenols which ultimately can alter the biological potential for modulating CVDs.

Moreover, there is increasing evidence that the activity and composition of the gut microbiota is implicated in many disease states, particularly in CVDs, in response to treatment [50]. Additionally, the composition of the gut microbiota strongly depends of diet [51,52,53,54] and plays a pivotal role on (poly)phenol metabolism [55,56,57].

To understand the impact of salt or the salt induced alterations in the metabolic fate of a (poly)phenol-enriched diet and gut microbiota composition, rats were fed a diet with or without salt or berries for 9 weeks. We used the Dahl/SS rat model, which is a validated chronic model of salt sensitive hypertension [58] mimicking the pathophysiological evolution of hypertension [59]. It was previously demonstrated for the same animals that the daily consumption of this berry mixture improved the survival, renal function, blood pressure and normalised the body weight of animals consuming a high-salt diet. In addition, a prophylactic effect was observed at the level of cardiac hypertrophy and dysfunction, tissue cohesion and cardiomyocyte hypertrophy [21]. Despite the beneficial effects observed it was unclear how berry components are absorbed, distributed, metabolised and excreted in these animals. The bioavailability and activity of (poly)phenols depend on diet, food matrices and interactions with other food constituents and while some food matrices can enhance their availability, others can reduce it [60,61]. The diets given to the rats were characterised by HPLC–MS in order to understand whether a high-salt content can influence the composition and release of the (poly)phenols from diet in vivo. HPLC–MS data revealed a great complexity and diversity of compounds present in the diets and demonstrated that the main factor that distinguishes the diets is the presence of berries and that salt does not interfere in the release of aglycones. Indeed, only three metabolites appeared to be significantly different (ANOVA) between high-salt berries and low-salt berries, which means that the presence of salt does not affect significantly the release of the aglycones from the food matrix.

Besides anthocyanins, which are the most abundant (poly)phenolic compounds present in our berry mixture [29], most of the metabolites found in urine and faeces were conjugated or non-conjugated phenolic acid derivatives. Only 6 of the metabolites are flavonoid derivatives, more specifically flavanols (catechin glucuronide, methyl-*O*-catechin sulphate, methyl-*O*-catechin glucuronide, epicatechin glucuronide and methyl-*O*-epicatechin sulphate).

The most abundant phenolic acid compound found at weeks 4 and 9 in urine and faeces was hippuric acid, followed by some hydroxycinnamic acids, and propionic acid derivatives. The majority of the compounds increased in urine from week 4 to week 9. This is in accordance with a prior study in humans that found a significant increase in the levels of *p*-coumaric acid and 3-hydroxyphenylacetic acid in urine after 8 weeks berry consumption [62]. Other studies using other sources of (poly)phenols, such as green tea, also report a significant increase in some urinary catechin metabolites, which is also in agreement with our results.

Cyanidin, the major anthocyanin present in our berry mixture, can be converted by the gut microbiota into benzaldehydes and small phenolic acids such as propionic, phenylacetic and some benzoic acids such as protocatechuic acid [63,64,65,66,67]. It is also reported that cyanidin can be the precursor to phloroglucinol, resorcinol, and pyrogallol [64]. These compounds can be sulphated and methylated or dehydroxylated, generating catechol [68] which, being a substrate for sulphatases, will increase the amount of catechol sulphate. These metabolites were detected in urine and plasma of human volunteers that consumed the same berry mixture that was given in this work to the rats [29,69]. The fact that cyanidin was the major anthocyanin present in the berry mixture, and its degradation products resulting from the gastro-intestinal digestion are phenolic acids, may explain the reason why phenolic acids are the most represented class of compounds that was found in urine and faeces of the animals used in this trial.

At week 4 of the diet, the urine samples of animals fed high concentrations of salt showed an increase in the release of the majority of the compounds (including anthocyanins), with the exception of some ferulic acid derivatives. By week 9 these effects were not as pronounced. However, after the chronic intake of salt those animals showed a decrease in the urinary excretion of some catechin derivatives. Animals fed salt enriched diets excreted lower amounts of ferulic acid derivatives regardless of the dietary supplementation with berries, meaning that the excretion rates of these compounds were affected in animals subjected to the salt-enriched diet. These compounds can result from the degradation of caffeoylquinic acids, which are also present in higher amounts in the berry mixture used in this trial [29]. Most of the caffeoylquinic acids arrive in the colon intact [55,70], where abundant esterases from colonic microbiota will hydrolyse the phenolic-quinic acid linkage [71]. Released phenolic acids are readily converted by the microbiota to the dihydro forms, such as dihydroferulic acid and 3-(dihydroxyphenyl) propionic acid, or can undergo phase II metabolism, namely sulphation and glucuronidation [72]. Dihydroferulic acid can be converted to vanillic acid [73] followed by phase II metabolism. Simpler phenolic acids, like the ones found in our study, such as gallic acid can undergo phase I and II metabolism, which will generate compounds such as pyrogallol [68], hippuric acid [63], syringic acid [74], gallic acid sulphate, and 4-*O*-methylgallic acid sulphate [69], also present in urine and faeces of the rats in this animal trial.

Moreover, Feliciano et al. hypothesised that peonidin present in higher amount in a cranberry juice given to humans, and also present in our berry mixture, could also lead to the formation of vanillic and ferulic acids [75] which is in agreement with our results.

Despite being present in all 4 groups, hippuric acid was excreted at higher amounts in urine and faeces of rats that consumed berries, particularly in the urine of HSB rats. These results are in agreement with previous studies where authors demonstrated that hippuric acid was significantly associated with blueberry and cranberry intake [75,76], a 12 week period of bilberry consumption [77], and with a high intake of a (poly)phenol enriched diet for 8 weeks [78]. This metabolite originates from the conjugation of colonic benzoic acids with glycine in the liver (amino acid metabolism) [61], which indicates that the presence of hippuric acid in LS and HS urines rats could be associated to other sources such as amino acid metabolism. This may also be true for other endogenous metabolites such as hydroxyphenylacetic acid, which can be derived from other sources than phenolic compounds.

Metabolite excretion in faeces at week 9 was lower than at week 4, which is in contrast to the pattern observed in urine samples. The faecal excretion of (poly)phenol metabolites was in the range of µmol/kg, and the majority of the ingested (poly)phenols are known to be transformed or degraded in the gut [79]. After 9 weeks of dietary intervention, the excretion of metabolites in the faeces was reduced suggesting that metabolism and absorption increased which could be associated with the increases detected in metabolite levels in urine. On the other hand, anthocyanins excretion was higher at the end of the trial as observed on both faecal and urine samples, especially for the animals consuming a salt-enriched diet. This high excretion rate is related with the low absorption of anthocyanins and hence their low bioavailability into the blood stream [64,80].

In order to evaluate the effects of a (poly)phenol enriched diet in the composition of gut microbiota and provide a link between bioactive metabolites of (poly)phenols and gut microbiota, we analysed the composition of the rat faecal microbiota over a period of 9 weeks. The faecal microbiota analysis clearly demonstrated that both the salt enriched diets and the consumption of berries, can induce changes in the microbiota and SCFA levels, two factors that have been implicated in the maintenance of a healthy gut physiology. This is in agreement with previous works showing that high-salt diet alters gut microbiota [81]. Despite no statistical differences in *Lactobacillus* species observed in our study, we observed a trend for increased *Lactobacillicaceae* in HS and HSB diets. Interestingly, a recent study with healthy male mice suggested that dietary salt increases blood pressure partly by affecting their gut microbiome, particularly by depleting *Lactobacilus murinus* [81]. Consequently, treatment with *L. murinus* prevented salt induced hypertension. It has also been demonstrated in a pilot study in humans that a moderate high-salt challenge reduced intestinal survival of *Lactobacillus* spp. and increased blood pressure [81]. Moreover, in a study conducted with the Dahl/SS rats, the authors demonstrated that these rats have a different microbiota than salt-resistant rats [82].

Furthermore, the consumption of (poly)phenol-rich diets has also been shown to have impacts on gut microbial population dynamics and gastro-intestinal tract health [83,84,85,86]. Lacombe et al., showed that lowbush wild blueberries had the potential to modify rat gut microbiota by decreasing the relative abundance of the genera *Lactobacillus* and *Enterococcus*, and increasing the relative abundance of the phylum Actinobacteria [83]. Moreover, in human volunteers, a six-week crossover dietary intervention demonstrated the potential of wild blueberries in modulating the intestinal microbiota. *Bifidobacterium* spp. significantly increased following blueberry consumption [86].

In our study, we demonstrate that members of the Bacteroidetes phylum increased due to the consumption of berries. Members of this phylum are versatile degraders of complex polysaccharides and plant compounds [87] and their increased abundance may be due to compounds present in the berries that support their growth. Firmicutes possess a disproportionately smaller number of glycan degrading enzymes than Bacteroidetes, and it might be hypothesised that intake of different (poly)phenols might reshape the gut microbiota [88], promoting the increase in Bacteroidetes as we observed in our study.

Moreover, we saw an increase in *Bacteroidaceae* family in rats fed a berry-enriched diet, which was also reported in other studies with fruit-enriched diets. For example, rats supplemented with apple fibre showed an increase in *Bacteroidaceae* family in almost one-log10 higher counts than in in faeces of control rats [89].

The consumption of berries also promoted an increase in the *S24-7* family in faecal samples from LSB and HSB rats, these are members of uncultured Bacteroidales that have been recently associated with the degradation of specific carbohydrates [90]. Differences in the abundance of *S24-7* family are associated with different environmental conditions, e.g., increased abundance in mice fed with cherries [91], diabetes-sensitive mice fed a high-fat diet [92], in rats with a high-salt diet [82] and following remission of colitis in a mouse model [93]. However, the metabolic functions and the consequences of these differences in abundance of *S24-7* are still unknown as they remain uncultured. However, genomic studies have been undertaken recently. In this study, we demonstrate for the first time that the consumption of berries has an impact on their abundance, as subsequent studies are necessary to elucidate their role in berry (poly)phenol metabolism.

Interestingly, the berry diet suppressed some changes in the microbiota caused by the high-salt diet such as the expansion of the phylum Proteobacteria and *Erysipelotrichaceae* family. There is strong evidence supporting an association between *Erysipelotrichaceae* and host lipid metabolism, warranting additional investigation into the metabolic profiles of these organisms, and the influence they have on the host [94]. Specific taxa within *Erysipelotrichaceae* have been correlated with inflammation [95], while others are highly immunogenic [96]. This highlights the importance of characterizing this bacterial family further which may eventually provide promising microbial targets to combat metabolic disorders. In our study, we found that the consumption of berries decreased the expansion of *Erysipelotrichaceae* promoted by the salt diet. A similar trend was observed in a study where treatment with quercetin attenuated the increase in Firmicutes/Bacteroidetes ratio in high-fat sucrose-diet-fed rats and significantly reduced the abundance of *Erysipelotrichaceae*, which is closely related to the consumption of Western diets [97].

SCFAs are the main fermentation product of gut microbiota and perform diverse functional roles that affect the host’s physiology, namely regulation of the blood pressure [98]. We investigated whether the faecal SCFA profiles changed due to the different diets. Only acetate was significantly different between groups. Acetate was elevated in the faeces of HS rats and the introduction of berries in the diet (HSB) promoted a significant decrease in the production of acetate. Though not statistically significant, the relative abundance of *Ruminococaceae* family was lower in the faeces of HS than in HSB rats. This family uses acetate to produce butyrate [99], and since they are present in lower abundance in HS faeces, they will consume less acetate thus explaining the SCFA accumulation in faeces. In contrast, *Ruminococaceae* family are more abundant in HSB rats where they consume acetate decreasing the amounts detected in faeces. The fact that SCFA measurements were conducted ex vivo in faeces does not reflect the overall production of SCFA. Rats are caecum fermenters and most of these compounds (95%–99%) are already absorbed in the large intestine. However, our work only accounts for the faecal SCFA excreted, which explains why only acetate is significantly altered.

Recent findings have demonstrated that changes in blood pressure are often coordinated with expected changes in SCFAs. One way that SCFAs can influence host cells is by interacting with host G-protein-coupled receptors (GPCRs), including GPR41, GPR43 and olfactory receptor 78 (Olfr78) [42] and by inducing host cell death [100]. SCFAs can stimulate the regulation pathways of GPCRs in order to affect renin secretion and therefore blood pressure [45,101]. In a study conducted with GPR41 knockout mice, these animals developed elevated systolic blood pressure when compared with wild-type mice, through the regulation of the endothelial GPR41 by the SCFA [102]. Moreover, propionate is a SCFA that can induce vasodilation and produce an acute hypotensive response in mice through modulation of Olfr78 and GPR41 activity [103]. Another study demonstrated that stimulation of GPR41 resulted in a reduction of the hypotensive response, and this effect could be mitigated by stimulating Olfr78 [45]. Additionally, the authors described that antibiotic treatment altered composition of gut microbiota and also increased blood pressure in Olfr78 knockout mice [45]. However, the details and intricacies of these interactions are not yet fully understood and will greatly benefit from further studies.

As the microbiota is thought to be important in mediating the effects of dietary salt [81,82] by promoting an increase in blood pressure either in animals and humans, it is possible that the effects of berry consumption by Dahl/SS rats on reducing the harmful effects of a high-salt diet are at least partially due to its ability to modify the gut microbiota. Further studies are important to uncover the main contributors for the beneficial effects of (poly)phenol consumption in this two-way interaction between (poly)phenols and gut microbiota: a specific gut microbial community associated with (poly)phenol metabolism, a specific microbial metabolite(s) produced, or both factors acting in an additive/synergistic manner [104].

## Figures and Tables

**Table 1:** (Poly)phenol metabolites in urine of rats fed a low salt (LS), low-salt supplemented with 2g of lyophilised berry mixture (LSB), high salt (HS) and high-salt supplemented with 2g of lyophilised berry mixture (HSB) at 4 and 9 weeks of trial. Results expressed in nmol as the mean ± SEM of values at 4 and 9 weeks of diet for six rats in the LS and LSB groups, for eight rats in the HS group and 12 rats in the HSB group.

	4th Week				9th Week			
	LS	LSB	HS	HSB	LS	LSB	HS	HSB
**Benzoic Acid Derivatives**								
Vanillic acid sulphate	15.3 ± 3.2	94.5 ± 7.4	6.9 ± 3.3	294.0 ± 21.4 ^###^	88.6 ± 35.9	276.0 ± 37.2	64.7 ± 27.8	288.0 ± 32.3 ^#^
Protocatechuic acid sulphate ^1^	51.8 ± 11.7	54.0 ± 8.8	605 ± 200	779 ± 217	90.6 ± 42.4	247.0 ± 48.7	1038 ± 120 **	702.0 ± 75.6
Hydroxybenzoic acid ^1^	32.7 ± 7.6	73.5 ± 10.6	206.0 ± 22.1 ***	313.0 ± 23.0 ^$$$^	134.0 ± 44.6	84.2 ± 25.8	173 ± 9.5	230 ± 34.7 ^$$$^
Hydroxybenzoic acid sulphate ^1^	n.d.	n.d.	2.58 ± 1.2	72.0 ± 8.1 ^$$$##^	6.2 ± 3.6	n.d.	138.0 ± 36.7 *	145.0 ± 14.9 ^$$$^
**Cinnamic Acid Derivatives**								
Coumaric acid sulphate ^1^	1.8 ± 0.6	117.0 ± 11.7 *	18.1 ± 2.3	329.0 ± 32.2 ^##^	n.d.	687 ± 143 ***	27.1 ± 3.3	679.0 ± 63.2 ^###^
Caffeic acid sulphate ^1^	7.0 ± 1.3	57.8 ± 5.0 ***	n.d.	143.0 ± 6.5 ^$$$###^	10.1 ± 3.5	130 ± 20.5 ***	n.d.	147.0 ± 8.6 ^###^
Caffeic acid glucuronide ^1^	1.13 ± 0.09	7.6 ± 0.9	n.d. *	13.3 ± 3.9 ^#^	n.d.	10.0 ± 2.2 **	n.d.	13.0 ± 3.4 ^#^
Ferulic acid	10.4 ± 0.8	31.8 ± 2.6	7.59 ± 1.76	61.9 ± 5.1 ^###^	9.4 ± 2.4	19.5 ± 3.8	n.d.	1.9 ± 0.8 ^$^
Ferulic acid sulphate	n.d.	31.2 ± 3.2 **	4.48 ± 2.43	35.9 ± 13.9	n.d.	87.1 ± 6.3 ***	n.d.	32.0 ± 7.8 ^#^
Ferulic acid glucuronide	0.65 ± 0.34	20.7 ± 1.5 ***	n.d.	4.2 ± 1.4 ^#^	n.d.	38.4 ± 7.8 ***	n.d.	2.4 ± 0.8 ^$^
**Catechol Derivatives**								
Catechol sulphate	n.d.	11.4 ± 7.1	436.0 ± 49.6 *	781.0 ± 55.4 ^$$$^	n.d.	730 ± 158 **	312 ± 55.4	1161 ± 175 ^$#^
4-methyl catechol sulphate ^1^	6.4 ± 2.9	4.7 ± 1.0	n.d.	22.6 ± 4.6 ^###^	19.8 ± 5.4	60.2 ± 8.4	21.1 ± 8.1	52.1 ± 7.8
4-Methyl catechol glucuronide ^1^	352 ± 111	221.0 ± 27.6	n.d. ***	240.0 ± 29.5 ^###^	n.d.	n.d.	n.d.	n.d.
**Pyrogallol Derivatives**								
Pyrogallol sulphate ^1^	40.2 ± 14.3	2.1 ± 1.3	576 ± 76.4 **	215 ± 33.9 ^$$^	n.d.	n.d.	n.d	n.d.
**Hippuric Acid Derivatives**								
Hippuric acid	734.0 ± 60.1	4225 ± 226 ***	1494 ± 96.0	13416 ± 937 ^###^	702 ± 135	4693 ± 332 *	862 ± 73.4	10609 ± 864 ^###^
**Propionic Acid Derivatives**								
3-(Hydroxyphenyl)propionic acid glucuronide ^1^	n.d.	0.09 ± 0.09	n.d.	n.d.	n.d.	5.4 ± 0.8 ***	5.4 ± 0.8	0.04 ± 0.04 ^$$$^
3-(4-hydroxyphenyl) propionic acid ^2^	36.5 ± 3.5	572 ± 75.5	177 ± 98.1	1357 ± 240 ^##^	4.16 ± 1.84	1347 ± 312 ***	15.7 ± 6.5	2068 ± 757 ^###^
3-(Dihydroxyphenyl) propionic acid ^1^	0.8 ± 0.2	14.5 ± 1.5	n.d.	137 ± 13.0 ^###^	n.d.	30.3 ± 4.7	31.5 ± 14.1	138 ± 25.4 ^###^
3-(Hydroxyphenyl)propionic acid sulphate ^1^	7.7 ± 1.7	206.0 ± 24.7	18.9 ± 4.9	1536 ± 180 ^###^	7.7 ± 1.7	1842 ± 336 **	32.2 ± 8.2	2975 ± 299 ^###^
Dihydroferulic acid	3.8 ± 0.3	19.7 ± 2.7	n.d.	2.1 ± 1.1 ^$$$^	3.1 ± 1.2	16.2 ± 1.9	0.16 ± 0.16	58.2 ± 10.7 ^###^
Dihydroferulic acid sulphate	3.3 ± 0.2	46.2 ± 5.8 **	n.d. *	5.6 ± 1.8 ^$$^	n.d.	52.5 ± 9.1 ***	n.d.	24.2 ± 5.5 ^##^
**Flavanols Derivatives**								
Methyl catechin glucuronide ^1^	1.1 ± 0.7	651.0 ± 54.4 ***	n.d.	528 ± 108 ^###^	n.d.	961 ± 138 ***	n.d.	407 ± 39.8 ^###^

* *p* < 0.05, ** *p* < 0.01, *** *p* < 0.001 versus LS; $ *p* < 0.05, $$ *p* < 0.01, $$$ *p* < 0.001 vs. LSB; # *p* < 0.05, ## *p* < 0.01, ### *p* < 0.001 vs. HS; n.d.: not detected; ^1^ it is possible to have more than one isomer for this compound that is not possible to distinguish by the MS/MS; ^2^ identity confirmed by standard.

**Table 2:** (Poly)phenol metabolites identified exclusively in urine of rats fed a LSB and HSB diet at 4 and 9 weeks of trial. Results are represented in nmol as the mean ± SEM of values at 4 and 9 weeks of diet for six rats in the LS and LSB groups, for eight rats in the HS group and 12 rats in the HSB group.

	4th Week		9th Week	
	LSB	HSB	LSB	HSB
**Benzoic Acid Derivatives**				
Vanillic acid	40.1 ± 12.7	n.d ^$^	85.9 ± 17.8 ***	19.0 ± 7.9 ^$^
Vanillic acid glucuronide ●	7.3 ± 2.4 ***	n.d. ^$$$^	n.d.	n..d
Gallic acid ●	n.d.	n..d	12.4 ± 7.3 **	n.d. ^$$$^
Gallic acid glucuronide ● ^1^	n.d.	n.d.	20.2 ± 8.6 ***	n.d. ^$$$^
4-*O*-methylgallic acid	88.6 ± 7.7 **	97.5 ± 8.7 ^###^	127.0 ± 10.7 ***	80.7 ± 13.3 ^$##^
4-*O*-methylgallic acid sulphate	38.7 ± 6.7	139.0 ± 11.8 ^###^	177.0 ± 36.5 **	204.0 ± 17.1 ^###^
Syringic acid ●	15.8 ± 1.7 ***	n.d. ^$$$^	21.8 ± 2.2 ***	n.d. ^$$$^
Syringic acid sulphate ●	2.5 ± 0.3 ***	n.d. ^$$$^	9.6 ± 0.8 ***	n.d. ^$$$^
**Cinnamic Acid Derivatives**				
Coumaric acid ^1^	18.7 ± 2.6	79.9 ± 6.8 ^###^	99.5 ± 18.7 **	99.9 ± 8.1 ^$$$^
Coumaric acid glucuronide ♦ ^1^	n.d.	1.8 ± 1.0	n.d.	5.8 ± 1.5 ^$$###^
**Phenylacetic Acids Derivatives**				
2-(Dihydroxyphenyl)acetic acid ^1^	2.1 ± 0.8	n.d.	5.4 ± 1.1 ***	4.9 ± 2.5
2-(Trihydroxyphenyl)acetic acid ^1^	31.9 ± 4.0 ***	25.6 ± 4.5 ^###^	33.7 ± 4.5 **	26.5 ± 5.9 ^##^
**Catechol Derivatives**				
Catechol glucuronide	n.d.	165.0 ± 7.9 ^$$$###^	46.8 ± 27.6	129.0 ± 21.7 ^$$$###^
**Valeric Acid Derivatives**				
5-(Hydroxyphenyl)valeric acid ^1^	2.8 ± 1.0 **	n.d. ^$$^	6.1 ± 2.2 ***	0.62 ± 0.44 ^$$^
**Valerolactone Derivatives**				
5-(Dihydroxypheny)l-γ-valerolactone ^1^	0.43 ± 0.22	n.d.	5.0 ± 1.2 *	17.1 ± 4.4 ^###^
5-(Dihydroxyphenyl)-γ-valerolactone sulphate ^1^	2.7 ± 0.9	3.1 ± 1.0	13.9 ± 1.2 **	23.3 ± 5.8 ^###^
5-(Dihydroxyphenyl)-γ-valerolactone glucuronide ● ^1^	n.d.	n.d.	0.97 ± 0.57 ***	n.d. ^$$$^
**Flavanols Derivatives**				
Catechin glucuronide ^1^	105.0 ± 9.8 ***	110.0 ± 19.3 ^###^	180 ± 19.4 ***	159.0 ± 24.1 ^###^
Methyl-*O*-catechin sulphate ^1^	0.24 ± 0.11	3.6 ± 1.8	8.7 ± 1.4	2.4 ± 1.2
Epicatechin glucuronide ^1^	56.4 ± 6.5 *	77.6 ± 11.0 ^###^	96.3 ± 23.9 **	99.2 ± 9.9 ^###^
Methyl-*O*-epicatechin sulphate ^1^	14.2 ± 2.7	51.4 ± 12.0 ^###^	30.2 ± 2.5 **	37.0 ± 13.2
Methyl-*O*-epicatechin glucuronide ^1^	114.0 ± 17.5 ***	67.7 ± 14.5 ^#^	221 ± 73.1 ***	67.3 ± 19.2

● Metabolites exclusively excreted in urine of LSB rats. ♦ Metabolites exclusively excreted in urine of HSB rats. * *p* < 0.05, ** *p* < 0.01, *** *p* < 0.001 versus LS; $ *p* < 0.05, $$ *p* < 0.01, $$$ *p* < 0.001 vs. LSB; # *p* < 0.05, ## *p* < 0.01, ### *i* < 0.001 vs. HS.; n.d.: not detected; ^1^ it is possible to have more than one isomer for this compound that is not possible to distinguish by the MS/MS.

**Table 3:** (Poly)phenol metabolites identified in faeces of the rats fed a LS, LSB, HS and HSB diet at 4 and 9 weeks of trial. Results expressed in μmol/kg fresh faeces as the mean ± SEM of values at 4 and 9 weeks of diet for six rats in the LS and LSB groups, for eight rats in the HS group and 12 rats in the HSB group.

	4th Week				9th Week			
	LS	LSB	HS	HSB	LS	LSB	HS	HSB
**Benzoic Acid Derivatives**								
Vanillic acid	1.3 ± 0.8	4.3 ± 1.7 **	n.d.	n.d.	n.d.	n.d.	n.d.	n.d.
Vanillic acid sulphate	3.7 ± 1.6	4.1 ± 1.8	n.d. **	n.d. ^$^	n.d.	n.d.	n.d.	n.d.
Gallic acid	16.6 ± 6.2	102.0 ± 25.1	n.d.	10.7 ± 0.8 ^###^	0.79 ± 0.55	12.5 ± 4.3	0.77 ± 0.49	3.5 ± 0.4
Gallic acid sulphate ^1^	48.9 ± 27.9	33.1 ± 14.6	n.d. **	n.d. ^$^	n.d.	n.d.	n.d.	n.d.
4-*O*-methyl gallic acid sulphate	1.2 ± 0.7	5.0 ± 2.3	n.d.	n.d. ^$^	n.d.	0.06 ± 0.05	n.d.	n.d.
Protocatechuic acid sulphate ^1^	8.0 ± 3.1	3.5 ± 1.4	n.d. ***	n.d.	0.32 ± 0.12	0.35 ± 0.14	n.d.	n.d.
Hydroxybenzoic acid ^1^	40.2 ± 15.3	17.7 ± 5.2	3.2 ± 1.2	3.6 ± 1.0	4.5 ± 1.2	4.4 ± 1.4	0.37 ± 0.24	3.5 ± 1.8
Hydroxybenzoic acid sulphate ^1^	51.2 ± 29.4	31.8 ± 12.2 **	n.d.	0.10 ± 0.07 ^$$^	0.00 ± 0.00	0.52 ± 0.26	n.d.	n.d.
**Cinnamic Acid Derivatives**								
Coumaric acid ^1^	3.5 ± 0.8	4.8 ± 1.9	0.34 ± 0.10	0.40 ± 0.07	n.d.	0.41 ± 0.15	n.d.	0.12 ± 0.08
Coumaric acid sulphate ^1^	14.1 ± 7.9	54.2 ± 19.9	n.d. **	2.1 ± 0.5 ^#^	n.d.	2.2 ± 0.6 *	n.d.	1.4 ± 0.3
Caffeic acid	27.6 ± 20.7	53.5 ± 24.4	n.d.	n.d. ^$$$^	n.d.	n.d.	n.d.	n.d.
Ferulic acid	7.2 ± 3.0	6.5 ± 2.9	0.12 ± 0.02	0.14 ± 0.03	0.28 ± 0.05	0.50 ± 0.16	0.02 ± 0.02	0.09 ± 0.05
Ferulic acid sulphate	10.1 ± 5.6	12.4 ± 5.3	n.d. *	n.d.	n.d.	0.35 ± 0.10 *	n.d.	n.d. ^$^
**Phenylacetic Acid Derivatives**								
2-(Phenyl)acetic acid	3039 ± 1541	1209 ± 225	87.4 ± 20.8	59.0 ± 16.0 ^$$^	117 ± 27.8	87.8 ± 15.9	38.1 ± 7.7	9.2 ± 2.6
2-(Hydroxyphenyl)acetic acid ^1^	197 ± 124	291.0 ± 37.3	7.0 ± 1.5 *	19.3 ± 3.8 ^$^	31.9 ± 5.3	51.1 ± 11.7	3.7 ± 0.6 *	8.2 ± 2.1
**Catechol Derivatives**								
Catechol sulphate	8.32 ± 1.46	7.26 ± 3.25	n.d. ***	0.41 ± 0.14	0.46 ± 0.17	0.71 ± 0.29	n.d.	0.19 ± 0.12
4-methyl catechol sulphate ^1^	5.65 ± 2.21	3.51 ± 1.38	n.d. *	0.01 ± 0.01	0.18 ± 0.12	0.16 ± 0.11	n.d.	n.d.
**Propionic acid Derivatives**								
3-(Phenyl)propionic acid	n.d.	3.3 ± 1.7	1.0 ± 0.3	2.3 ± 0.3	2.7 ± 0.9	3.2 ± 1.0	1.2 ±0.4	1.4 ± 0.3
3-(hydroxyphenyl) propionic acid ^1^	55.0 ± 17.9	353 ± 125	2.7 ± 0.4	10.0 ± 2.3	n.d.	53.7 ± 12.1	0.87 ± 0.55	5.9 ± 1.7 ^$$$^
(Hydroxyphenyl)propionic acid sulphate ^1^	62.1 ± 35.2	299 ± 128	n.d. **	0.66 ± 0.23	10.0 ± 4.3	11.6 ± 3.8	n.d.	0.20 ± 0.13
Dihydroferrulic acid	4.2 ± 2.7	3.2 ± 1.2	n.d.	n.d. ^$^	0.28 ± 0.08	0.39 ± 0.16	n.d.	n.d.
**Hippuric Acid Derivatives**								
Hippuric acid	546 ± 326	1567 ± 701	0.41 ± 0.27 **	2.9 ± 0.5	2.4 ± 0.6	64.9 ± 21.4	0.48 ± 0.30	0.94 ± 0.39 ^$$^

* *p* < 0.05, ** *p* < 0.01, *** *p* < 0.001 versus LS; $ *p* < 0.05, $$ *p* < 0.01, $$$ *p* < 0.001 vs. LSB; # *p* < 0.05, ## *p* < 0.01, ### *p* < 0.001 vs. HS; n.d.: not detected; ^1^ It is possible to have more than one isomer for this compound that is not possible to distinguish by the MS/MS.

**Table 4:** (Poly)phenol metabolites exclusively identified in faeces of the rats fed a LSB and HSB diet at 4 and 9 weeks of trial. Results are represented in μmol/kg fresh faeces as the mean ± SEM of values at 4 and 9 weeks of diet for six rats in the LS and LSB groups, for eight rats in the HS group and 12 rats in the HSB group.

	4th Week			9th Week	
	LSB		HSB	LSB	HSB
**Benzoic Acid Derivatives**					
Protocatechuic acid	73.2 ± 22.2 ***		6.6 ± 1.2 ^###^	n.d.	n.d.
4-*O*-methyl gallic acid ●	10.0 ± 1.3 ***		n.d. ^$$$^	1.7 ± 0.2 **	n.d. ^$$^
Syringic acid	19.5 ± 2.6 ***		2.8 ± 0.5 ^##^	4.5 ± 0.8 **	1.6 ± 0.3
**Propionic Acid Derivatives**					
3-(dihydroxyphenyl) propionic acid ● ^1^	n.d.		n.d.	1.5 ± 0.8 **	n.d. ^$$^
**Valeric Acid Derivatives**					
5-(Hydroxyphenyl)valeric acid ^1^	84.9 ± 16.8 ***		3.9 ± 0.7 ^#^	25.0 ± 9.2 ***	1.7 ± 0.5
**Flavanols Derivatives**					
Catechin	n.d.		1.7 ± 0.6	0.34 ± 0.23	6.5 ± 4.2 ^#^
Epicatechin	13.0 ± 8.5		7.1 ± 2.4 ^###^	n.d.	3.3 ± 0.9
Diarylpropan-2-ol ^1^	2.9 ± 1.3		0.22 ± 0.15	0.94 ± 0.37 *	0.21 ± 0.14

● Metabolites exclusively excreted in faeces of LSB rats. * *p* < 0.05, ** *p* < 0.01, *** *p* < 0.001 versus LS; $$ *p* < 0.01, $$$ *p* < 0.001 vs. LSB; # *p* < 0.05, ## *p* < 0.01, ### *p* < 0.001 vs. HS; n.d.: not detected; ^1^ It is possible to have more than one isomer for this compound that is not possible to distinguish by the MS/MS.

## Supplementary Materials

The following are available online at https://www.mdpi.com/2072-6643/11/11/2634/s1, Figure S1—Total ion chromatograms of non-hydrolysed HSB, LSB, HS and LS diet extracts acquired in ESI positive mode (**A**) and ESI negative mode (**B**); Figure S2: Impact of salt and berry supplementation in the composition of the diets. HPLC–MS (negative and positive mode) data of (A) non-hydrolysed samples and (B) hydrolysed samples was processed by means of a Principal Component Analysis (PCA). N = 3. Samples clustering is indicated; Supplementary Figure S3—Total ion chromatograms of hydrolysed HSB, LSB, HS and LS diet extracts acquired in ESI positive mode (A) and ESI negative mode (B); Figure S4: Berries improve body weight and blood pressure in Dahl-salt sensitive rats. (A) Serial data on body weight. Data represents the mean ± S.E.M. * *p* < 0.05 vs. LS; $ *p* < 0.05 vs. LSB; # *p* < 0.05 vs. HS. N = 6 LS, N = 6 LSB, N = 8 HS, N = 13 HSB. (C) SBP measurement by tail cuff method. Data represents mean ± S.E.M. * *p* < 0.05 vs. LS; $ *p* < 0.05 vs. LSB; # *p* < 0.05 vs. HS. N = 6 LS, N = 6 LSB, N = 8 HS. N = 13 HSB. This animal trial was also used for study the cardioprotective mechanisms, and therefore this data was also presented in a preceding paper published in Oudot C. et al., Nutritional Biochemistry, doi:10.1016/j.jnutbio.2019.01.001 (2019); Table S1: Optimised SRM conditions for analysing the phenolic compounds determined in urine and faeces samples by UPLC–MS/MS; Table S2: Proximate composition of diets was determined based on the standard methods of the Association of Official Analytical Chemists (AOAC); Table S3: Proximate composition of berry mixture was determined based on the standard methods of the Association of Official Analytical Chemists (AOAC); Table S4: List of tentatively annotated metabolites present in the chemical analysis in ESI positive mode of the different non-hydrolysed diets. Analysis of variance results comparing berry diets with either high salt or low salt composition include significance level (F pr.), grand mean, high salt mean, low salt mean and standard error of means (SEM) for each metabolite. Identification level corresponds to the levels of confidence on the annotation of the metabolite: 1—Annotation based on two or more orthogonal properties with an authentic chemical standard analysed under identical analytical conditions; 2—based upon physicochemical properties and/or spectral similarity with public commercial spectral libraries, without reference to authentic chemical standards; 3—based upon characteristic physicochemical properties of a chemical class of compounds, or by spectral similarity to know compounds of a chemical class; 4—unidentified and unclassified, these metabolites can still be differentiated and quantified based upon spectral data; Table S5: List of tentatively annotated metabolites present in the chemical analysis in ESI negative mode of the different non-hydrolysed diets. Analysis of variance results comparing berry diets with either high salt or low salt composition include significance level (F pr.), grand mean, high salt mean, low salt mean and standard error of means (SEM) for each metabolite. Identification level corresponds to the levels of confidence on the annotation of the metabolite: 1–Annotation based on two or more orthogonal properties with an authentic chemical standard analysed under identical analytical conditions; 2—based upon physicochemical properties and/or spectral similarity with public commercial spectral libraries, without reference to authentic chemical standards; 3—based upon characteristic physicochemical properties of a chemical class of compounds, or by spectral similarity to know compounds of a chemical class; 4—unidentified and unclassified, these metabolites can still be differentiated and quantified based upon spectral data; Table S6—Characterization and quantification of anthocyanins in the berry mixture. Anthocyanins were analysed by UPLC–MS/MS. The analytical column was Acquity BEH UPLC (100 × 2.1 mm, 1.7 µm), the mobile phase was 10% acetic acid (eluent A) and acetonitrile (eluent B) [26]. The injection volume was 2.5 µL and the flow rate was 0.3 mL/min; Table S7: Characterization and quantification of main aglycones in the berry mixture, after enzymatic hydrolysis using HPLC-DAD relative to authentic standards as previously described [60]; Table S8: List of tentatively annotated metabolites present in the chemical analysis in ESI positive mode of the different hydrolysed diets. Analysis of variance results comparing berry diets with either high salt or low salt composition include significance level (F pr.), grand mean, high salt mean, low salt mean and standard error of means (SEM.) for each metabolite. Identification level corresponds to the levels of confidence on the annotation of the metabolite: 1—Annotation based on two or more orthogonal properties with an authentic chemical standard analysed under identical analytical conditions; 2—based upon physicochemical properties and/or spectral similarity with public commercial spectral libraries, without reference to authentic chemical standards; 3—based upon characteristic physicochemical properties of a chemical class of compounds, or by spectral similarity to know compounds of a chemical class; 4—unidentified and unclassified, these metabolites can still be differentiated and quantified based upon spectral data; Table S9: List of tentatively annotated metabolites present in the chemical analysis in ESI negative mode of the different hydrolysed diets. Analysis of variance results comparing berry diets with either high salt or low salt composition include significance level (F pr.), grand mean, high salt mean, low salt mean and standard error of means (SEM) for each metabolite. Identification level corresponds to the levels of confidence on the annotation of the metabolite: 1—Annotation based on two or more orthogonal properties with an authentic chemical standard analysed under identical analytical conditions; 2—based upon physicochemical properties and/or spectral similarity with public commercial spectral libraries, without reference to authentic chemical standards; 3—based upon characteristic physicochemical properties of a chemical class of compounds, or by spectral similarity to know compounds of a chemical class; 4—unidentified and unclassified, these metabolites can still be differentiated and quantified based upon spectral data.
