# Supplementary material for: Berry-Enriched Diet in Salt-Sensitive Hypertensive Rats: Metabolic Fate of (Poly)Phenols and the Role of Gut Microbiota
Source: Nutrients. 2019 Nov 3;11(11):2634. doi: 10.3390/nu11112634 (PMC6893819; doi:10.3390/nu11112634)
Supplement: Supplementary file 1 [file nutrients-11-02634-s001.pdf]

Article

Berry-enriched diet in salt-sensitive hypertensive rats: metabolic fate of (poly)phenols and the role of gut microbiota

Andreia Gomes[1,2], Carole Oudot[3], Alba Macià[4], Alexandre Foito[5], Diogo Carregosa[1,2,6], Derek Stewart[5,7], Tom Van de Wiele[8], David Berry[9], Maria-José Motilva[4,10], Catherine Brenner[3], Cláudia Nunes dos Santos[1,2,6]

[1]Instituto de Biologia Experimental e Tecnológica, Apartado 12, 2780-901 Oeiras, Portugal; andreia.gomes@nms.unl.pt (AG)
[2]Instituto de Tecnologia Química e Biológica, Universidade Nova de Lisboa, Av. da República, 2780-157 Oeiras, Portugal;
[3]INSERM UMR-S 1180- University Paris-Sud, University Paris Saclay, 5 rue Jean-Baptiste Clément 92296 Châtenay Malabry, France; oudot.carole@gmail.com (CO); catherinebrenner@yahoo.com (CB)
[4]Food Technology Department, Agrotecnio Center, Escuela Técnica Superior de Ingeniería Agraria, University of Lleida, Lleida, Spain; albamacia@tecal.udl.cat (AM)
[5]Environmental and Biochemical Sciences, James Hutton Institute, Invergowrie, Dundee DD2 5DA, Scotland, UK; alex.foito@hutton.ac.uk (AF); derek.Stewart@hutton.ac.uk (DS)
[6]Centro de Estudos de Doenças Crónicas (CEDOC), Rua Câmara Pestana nº 6, 6-A, Edifício CEDOC II, 1150-082 Lisboa; diogo.carregosa@nms.unl.pt (DC); claudia.nunes.santos@nms.unl.pt (CNS)
[7]Institute of Mechanical Process and Energy Engineering, School of Engineering and Physical Sciences, Heriot Watt University, Edinburgh, Scotland, UK;
[8]Center for Microbial Ecology and Technology (CMET), Ghent University, Coupure Links 653, 9000, Ghent, Belgium; Tom.VandeWiele@ugent.be (TVW)
[9]Division of Microbial Ecology, Department of Microbiology and Ecosystem Science, Research Network Chemistry Meets Microbiology, University of Vienna, Althanstr. 14 A-1090 Vienna, Austria; berry@microbial-ecology.net (DB)
[10]Instituto de Ciencias de la Vid y del Vino-ICVV, CSIC-Universidad de La Rioja-Gobierno de La Rioja, Finca “La Grajera”, Carretera de Burgos km 6, 26007-Logroño, Spain; motilva@icvv.es (MJM)

*Correspondence: claudia.nunes.santos@nms.unl.pt; Tel.: +351 218 803 101

Field Code Changed

Field Code Changed

Field Code Changed

Field Code Changed

Field Code Changed

Field Code Changed

Formatted: Portuguese (Portugal)

Supplementary Figure S1 - Total ion chromatograms of non-hydrolysed HSB, LSB, HS and LS diet extracts acquired in ESI positive mode (A) and in ESI negative mode (B).

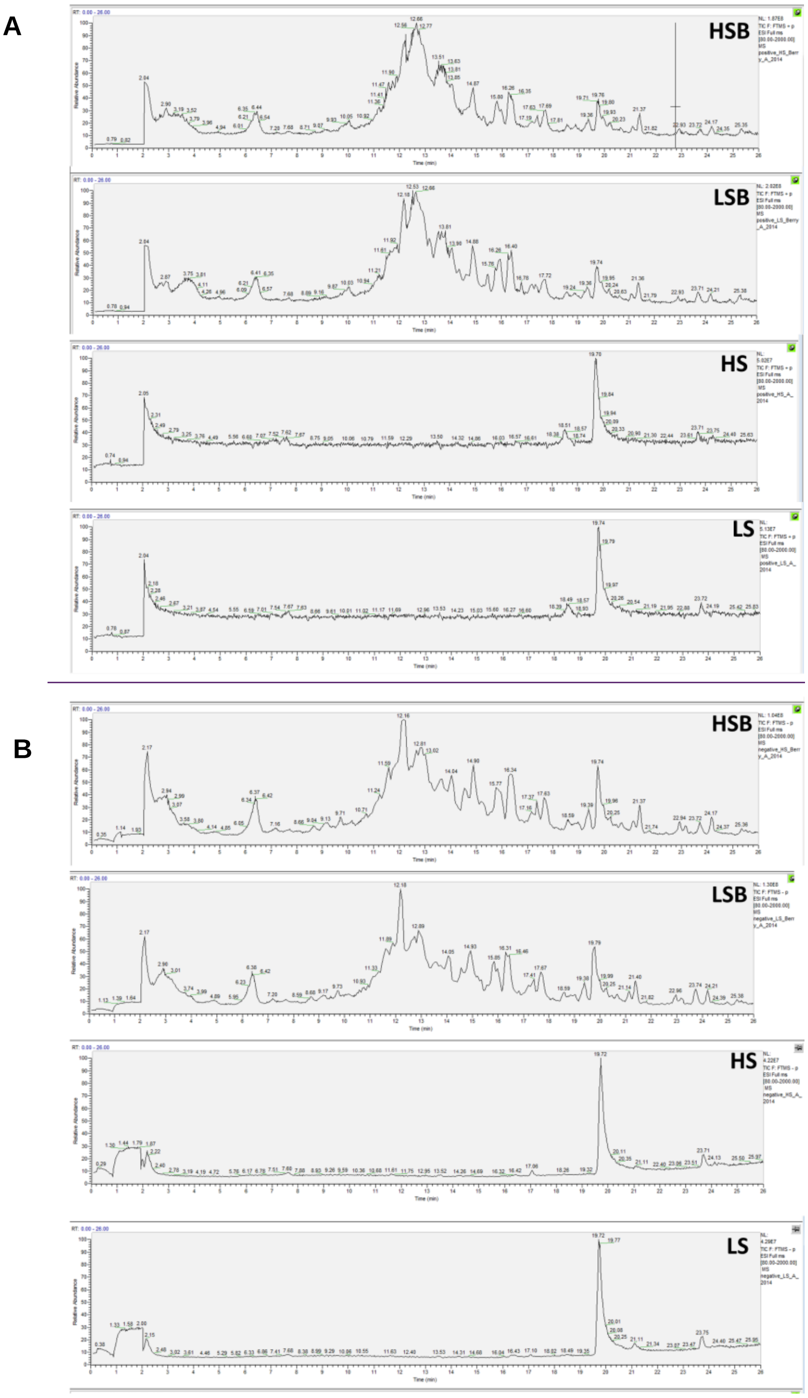

Supplementary Figure S2~~1~~ - Impact of salt and berry supplementation in the composition of the diets. HPLC-MS (negative and positive mode) data of A) non-~~hydrolyzed~~hydrolysed samples and B) ~~hydrolyzed~~hydrolysed samples were processed by means of a Principal Component Analysis (PCA). N=3. Samples clustering is indicated.

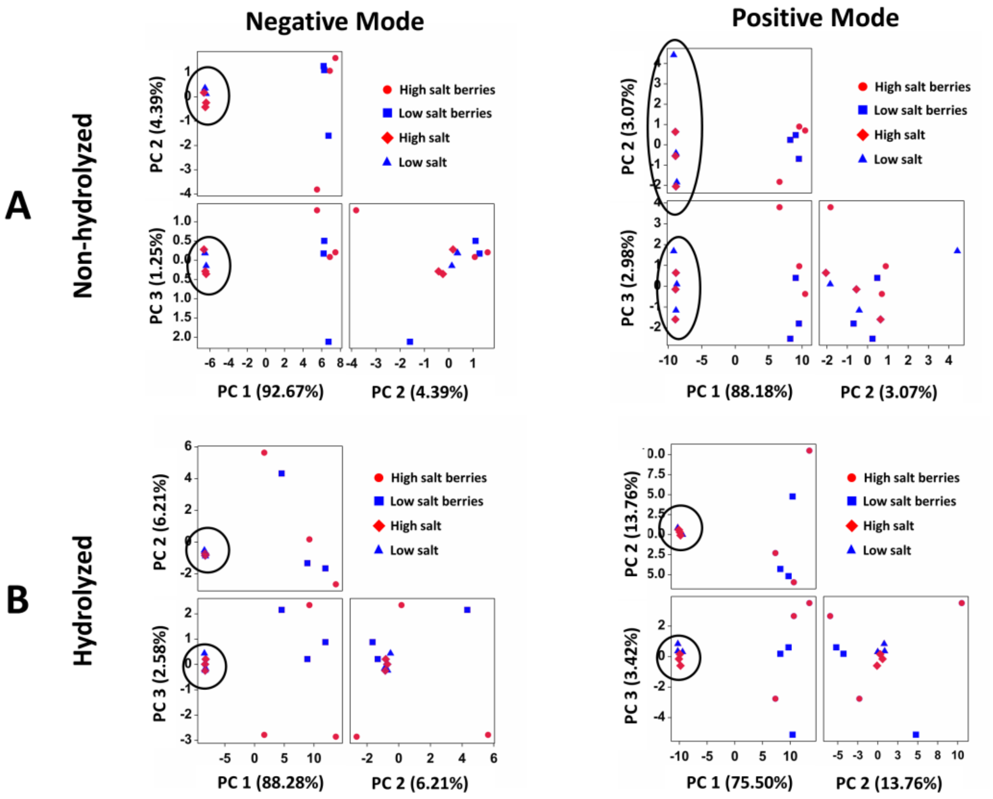

Supplementary Figure S3 - Total ion chromatograms of hydrolysed HSB, LSB, HS and LS diet extracts acquired in ESI positive mode (A) and ESI negative mode (B).

A

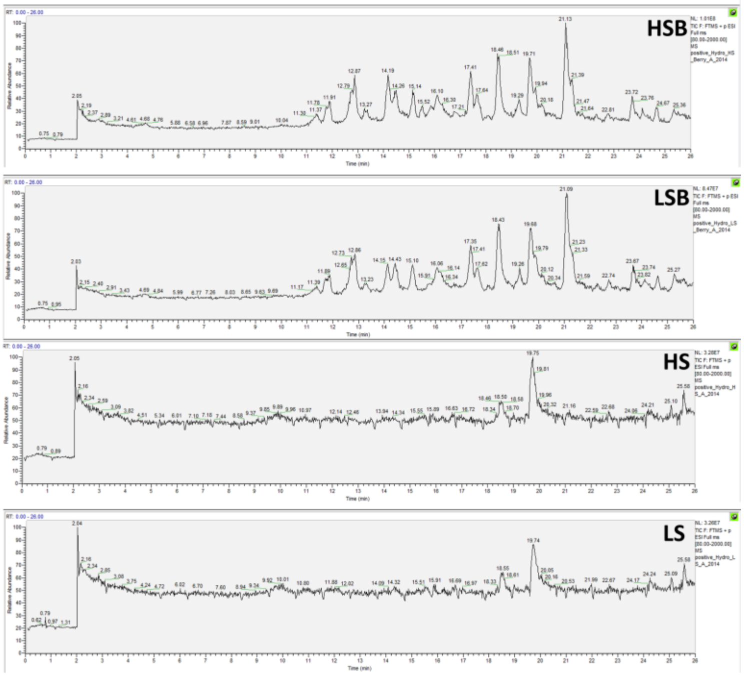

B

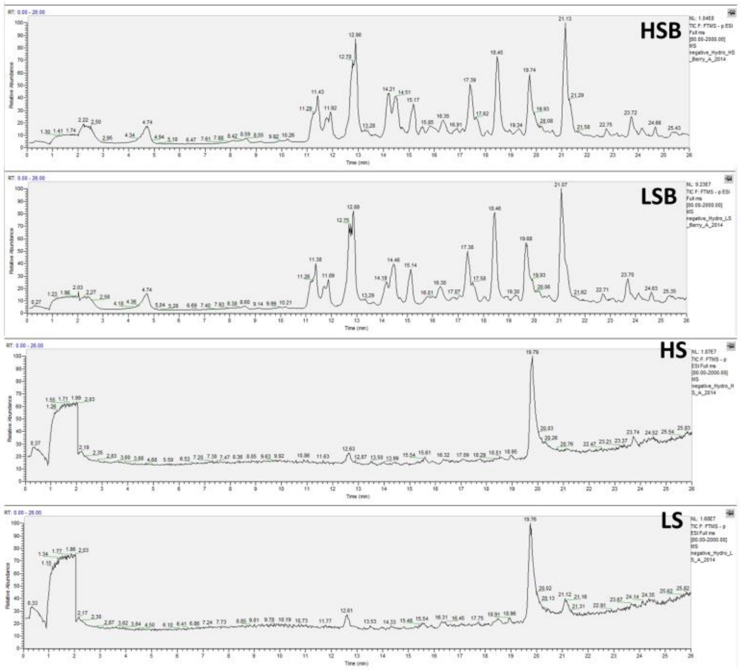

Supplementary Figure S~~2~~4 - Berries improve body weight and blood pressure in Dahl-salt sensitive rats. ((A) Serial data on body weight. Data represent mean±S.E.M. *p<0.05 vs. LS; $P<.05 vs. LSB; #P<.05 vs. HS. N=6 LS, N=6 LSB, N=8 HS, N=13 HSB. (C) SBP measurement by tail cuff method. Data represent mean±S.E.M. *p<0.05 vs. LS; $P<.05 vs. LSB; #P<.05 vs. HS. N=6 LS, N=6 LSB, N=8 HS. N=13 HSB. This animal trial was also used for study the cardioprotective mechanisms, therefore this data was also presented in a preceding paper published in Oudot C. *et al*., Nutritional Biochemistry, doi: 10.1016/j.jnutbio.2019.01.001 (2019).

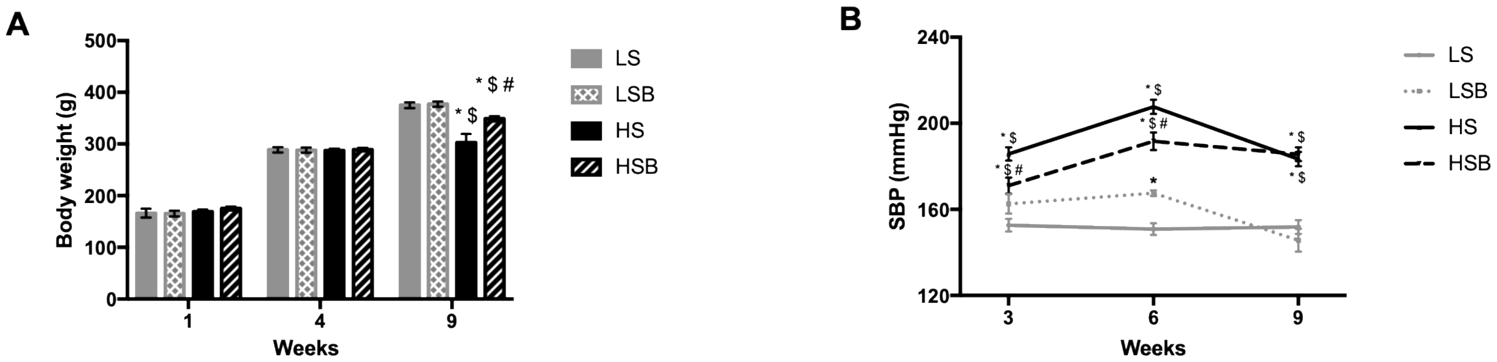

Supplementary Table S1 - Optimized SRM conditions for analysing the phenolic compounds determined in urine and faeces samples by UPLC-MS/MS.

Phenolic compound	SRM quantification			SRM identification		
	Transition	Cone voltage (V)	Collision energy (eV)	Transition	Cone voltage (V)	Collision energy (eV)
**Phenolic acids**						
Hydroxybenzoic acids						
Hydroxybenzoic acid	137 > 93	30	15	-	-	-
Hydroxybenzoic acid sulfate	217 > 137	35	15	-	-	-
Hippuric acid	178 > 134	40	15	-	40	25
Gallic acid	169 > 125	35	10	169 > 97	35	15
Gallic acid sulfate	249 > 169	35	10	249 > 125	35	25
Gallic acid glucuronide	345 > 169	35	15	345 > 125	35	25
4-*O*-methyl gallic acid	183 > 125	40	15	-	-	-
Methyl gallic acid sulfate	263 > 183	40	15	263 > 125	40	25
Protocatechuic acid	153 > 109	45	15	-	-	-
Protocatechuic acid sulfate	233 > 109	45	20	-	-	-
Syringic acid	197 > 182	30	10	197> 153	30	10
Syringic acid sulfate	277 > 197	35	10	277 > 182	30	20
Hydroxycinnamic acids						
Caffeic acid	179 > 135	35	15	179 > 117	35	20
Caffeic acid sulfate	259 > 179	35	15	259 > 135	35	25
Caffeic acid glucuronide	355 > 179	40	15	355 > 135	40	20
Vanillic acid	167 > 123	30	10	167 > 152	30	15
Vanillic acid sulfate	247 > 167	30	10	247 > 152	30	15
Vanillic acid glucuronide	343 > 167	30	10	343 > 152	30	15
p-Coumaric acid	163 > 119	25	10	163 > 117	25	25
Coumaric acid sulfate	243 > 163	40	20	243 > 119	40	25
Coumaric acid glucuronide	339 > 163	40	20	339 > 119	40	25
Ferulic acid	193 > 134	30	15	193> 178	30	10
Ferulic acid sulfate	273 > 193	35	15	273 > 134	30	25
Ferulic acid glucuronide	369 > 193	35	15	369 > 134	30	20
**Phenylacetic acids**						
Phenylacetic acid	135 > 91	20	5	-	-	-
4-Hydroxyphenylacetic acid	151 > 107	20	10	-	-	-
3,4-Dihydroxypheylacetic acid	167 > 123	20	10	-	-	-
2,4,5-Trihydroxypheylacetic acid	183 > 139	20	10	-	-	-
**Phenylpropionic acids**						
2-Phenylpropionic acid	149 > 105	20	5	-	-	-
3-(4-hydroxyphenyl) propionic acid	165 > 121	20	10	-	-	-
Hydroxyphenyl propionic acid sulfate	245 > 165	20	10	245 > 121	20	10
Hydroxyphenyl propionic acid glucuronide	341 > 165	20	10	341 > 121	20	10
3-(2,4-dihydroxyphenyl) propionic acid (dihydrocaffeic acid)	181 > 137	20	15	-	-	-
3-(4-hydroxy-3-methoxyphenyl) propionic acid (dihydroferulic acid)	195 > 135	40	25	-	-	-
Hydroxymethoxyphenyl propionic acid sulfate (dihydroferulic acid sulfate)	275 > 195	40	25	275 > 135	40	25
**Flavan-3-ols**						
(epi)catechin	289 > 245	45	15	289 > 179	45	15
Methyl (epi)catechin sulfate	383 > 303	40	15	383 > 289	45	25
(Epi)catechin glucuronide	465 > 289	40	20	465 > 245	45	25
Methyl (epi)catechin glucuronide	479 > 303	40	25	479 > 289	45	25
Diarylpropan-2-ol	275 > 107	40	25	-	-	-
**Valeric acid derivatives**						
Hydroxyphenylvaleric acid	193 > 149	40	15	-	-	-
**Valerolactone derivatives**						
Hydroxyphenyl-γ-valerolactone	191 > 147	40	10	-	-	-
Dihydroxyphenyl-γ-valerolactone	207 > 163	40	10	-	-	-
Dihydroxyphenyl-γ-valerolactone sulfate	287 > 207	40	15	287 > 163	40	25

Dihydroxyphenyl-γ-valerolactone glucuronide	383 > 207	40	20	383 > 163	40	25
**Cathecol derivatives**						
Catechol sulfate	189 > 109	40	15	-	-	-
Catechol glucuronide	285 > 109	40	20	-	-	-
4-*O*-methyl catechol sulfate	203 > 123	40	15	-	-	-
4-*O*-methyl catechol glucuronide	299 > 123	40	15	-	-	-
**Pyrogallol derivatives**						
Pyrogallol sulfate	205 > 125	40	15	-	-	-

Supplementary Table S2 - Proximate composition of diets was determined based on the standard methods of the Association of Official Analytical Chemists (AOAC).

	**LS**	**LSB**	**HS**	**HSB**
**Protein (g/100g)**	15.00 ± 0.04	13.80 ± 0.55	15.30 ± 0.28	13.40 ± 0.27
**Lipids (g/100g)**	3.79 ± 0.004	3.54 ± 0.16	3.32 ± 0.01	3.54 ± 0.20
**Water (g/100g)**	15.60 ± 0.07	18.00 ± 0.12	17.50 ± 0.22	18.50 ± 0.08
**Fibres (mg/100g)**	1.83 ± 0.01	3.02 ± 0.12	3.03 ± 0.48	2.47 ± 0.22
**Ashes (mg/100g)**	2.56 ± 0.17	2.91 ± 0.24	8.99 ± 0.17	8.49 ± 0.04
**Carbohydrates (g/100g)**	61.40 ± 0.32	58.70 ± 0.71	51.90 ± 0.27	55.00 ± 1.60

Values are represented as: mean ± SEM; LS: Low Salt; HS: High Salt; LSB: Low Salt Berries; HSB: High Salt Berries; N=3.

Supplementary Table S3 - Proximate composition of berry mixture was determined based on the standard methods of the Association of Official Analytical Chemists (AOAC).

	**2g of lyophilized Berry Mixture***
**Energy (kJ)**	4.74
**Energy (kcal)**	1.13
**Protein (g)**	0.08
**Lipids (g)**	0.04
**Water (g)**	0.25
**Fibres (mg)**	0.37
**Ashes (mg)**	0.04
**Carbohydrates (g)**	1.21
**D-Glucose (g)**	0.04
**D-Fructose (g)**	0.05
**Sucrose (g)**	0.01

Values are represented as: mean; N=3. *2 g of lyophilized berries added to 50g of diet.

Supplementary Table S4 - List of tentatively annotated metabolites present in the chemical analysis in ESI positive mode of the different non-hydrolysed diets. Analysis of variance results comparing berry diets with either high salt or low salt composition include significance level (F pr.), grand mean, high salt mean, low salt mean and standard error of means (SEM) for each metabolite. Identification level corresponds to the levels of confidence on the annotation of the metabolite: 1- Annotation based on two or more orthogonal properties with an authentic chemical standard analysed under identical analytical conditions; 2 - based upon physicochemical properties and/or spectral similarity with public commercial spectral libraries, without reference to authentic chemical standards; 3 - based upon characteristic physicochemical properties of a chemical class of compounds, or by spectral similarity to know compounds of a chemical class; 4 - unidentified and unclassified, these metabolites can still be differentiated and quantified based upon spectral data.

Compound	Retention Time	m/z	adduct	ms2	Molecular Formula	Identification level	F pr.	Grand mean	High salt mean	Low salt mean	SEM
Galloyl-quinic acid isomer A	3.22	345.0816	[M+H]+	153; 327	$C_{14}H_{16}O_{10}$	2	0.794	9.00E+08	9.00E+08	9.00E+08	47731098.9
Galloyl-quinic acid isomer B	6.44	345.0816	[M+H]+	153; 327	$C_{14}H_{16}O_{10}$	2	0.93	7.00E+08	6.00E+08	7.00E+08	5.95E+07
Pantothenic acid isomer A	7.02	220.1179	[M+H]+	202; 90; 184	$C_9H_{17}NO_5$	2	0.257	52371783	4.00E+07	6.00E+07	1.19E+07
Pantothenic acid isomer B	7.65	220.1179	[M+H]+	202; 90; 184	$C_9H_{17}NO_5$	2	0.177	5.63E+07	5.00E+07	6.00E+07	9.03E+06
Gallocatechin	8.89	307.0813	[M+H]+	139; 289; 151	$C_{15}H_{14}O_7$	1	0.621	2.11E+07	2.00E+07	2.00E+07	4.08E+06
Gallocatechin-epicatechin isomer A	9.05	595.1446	[M+H]+	443; 287; 425; 291; 317	$C_{30}H_{26}O_{13}$	2	0.584	8.05E+06	7.95E+06	8.15E+06	3.40E+05
Epigallocatechin	9.17	307.0813	[M+H]+	-	$C_{15}H_{14}O_7$	1	0.766	3.26E+07	3.00E+07	3.00E+07	5.73E+06
Galloyl-shikimic acid isomer A	9.19	327.0711	[M+H]+	153; 309; 171; 251; 139; 291	$C_{14}H_{14}O_9$	2	0.862	5.68E+06	6.11E+06	5.24E+06	4.67E+06
Unknown (A)	9.3	624.2133	[M+H]+	300; 606; 264; 462; 325; 198	$C_{25}H_{37}NO_{17}$	4	0.965	5.66E+06	5.64E+06	5.68E+06	8.88E+05
Galloyl-shikimic acid isomer B	9.46	327.0711	[M+H]+	-	$C_{14}H_{14}O_9$	2	0.941	4.47E+07	4.00E+07	4.00E+07	7.19E+06
Galloyl-shikimic acid isomer C	9.66	327.0711	[M+H]+	-	$C_{14}H_{14}O_9$	2	0.902	5.25E+07	5.00E+07	5.00E+07	5.59E+06
Gallocatechin-epicatechin isomer B	9.87	595.1445	[M+H]+	443; 427; 425; 291; 317; 287	$C_{30}H_{26}O_{13}$	2	0.947	3.45E+07	3.00E+07	3.00E+07	2.50E+06
Neochlorogenic acid isomer A	9.93	355.1024	[M+H]+	-	$C_{16}H_{18}O_9$	1	0.334	2.28E+07	2.00E+07	2.00E+07	1.76E+06
Neochlorogenic acid isomer B	10.11	355.1024	[M+H]+	-	$C_{16}H_{18}O_9$	1	0.764	4.28E+07	4.00E+07	4.00E+07	5.40E+06
Chlorogenic acid glucoside	10.42	517.155	[M+H]+	-	$C_{22}H_{28}O_{14}$	3	0.874	1.23E+07	1.00E+07	1.00E+07	2.54E+06
Dihydro-caffeic acid glucuronide	10.51	359.0973	[M+H]+	167; 341	$C_{15}H_{18}O_{10}$	3	0.189	2.03E+07	2.00E+07	2.00E+07	4.37E+06

Unknown (B)	10.71	462.1605	[M+H]+	138; 325; 300; 444; 163; 264; 282	$C_{19}H_{27}NO_{12}$	3	0.945	2.06E+07	2.00E+07	2.00E+07	2.90E+06
Gallocatechin-catechin-catechin	10.84	883.2079	[M+H]+	-	$C_{45}HO_{19}$	3	0.898	9.96E+06	1.00E+07	9.91E+06	6.24E+05
Neochlorogenic acid dimer	10.98	709.1973	[M+H]+	691; 499; 355; 517	$C_{32}H_{36}O_{18}$	3	0.754	7.92E+07	8.00E+07	8.00E+07	9.42E+06
Procyanidin B isomer A	11.21	579.1495	[M+H]+	427; 409; 291; 247; 301	$C_{30}H_{26}O_{12}$	2	0.758	5.06E+07	5.00E+07	5.00E+07	6.41E+06
Procyanidin B isomer B	11.47	579.1495	[M+H]+	-	$C_{30}H_{26}O_{12}$	2	0.295	4.35E+07	4.00E+07	4.00E+07	2.02E+06
Delphinidin 3-O-glucoside	11.72	465.1024	[M]+	303	$C_{21}H_{21}O_{12}^{+}$	1	0.753	2.00E+09	2.00E+09	2.00E+09	7.77E+07
Catechin	11.9	291.0863	[M+H]+	139; 123; 165; 151; 273	$C_{15}H_{14}O_6$	1	0.96	2.00E+08	2.00E+08	2.00E+08	7.05E+06
Cyanidin 3-O-sophoroside	12.08	611.1605	[M]+	287	$C_{27}H_{31}O_{16}$+	1	0.835	1.00E+08	1.00E+08	1.00E+08	9.33E+06
Chlorogenic acid	12.14	355.1024	[M+H]+	163	$C_{16}H_{18}O_9$	1	0.884	8.00E+08	8.00E+08	8.00E+08	3.92E+07
Cyanidin 3-O-glucosyl-rutinoside	12.31	757.2181	[M]+	287	$C_{33}H_{41}O_{20}$+	2	0.634	2.00E+08	2.00E+08	2.00E+08	1.32E+07
Cyanidin 3-O-glucoside	12.56	449.1075	[M]+	287	$C_{21}H_{21}O_{11}$+	1	0.863	3.00E+09	3.00E+09	3.00E+09	1.55E+08
Cyanidin 3-O-rutinoside	12.92	595.1655	[M]+	287	$C_{27}H_{31}O_{15}$+	1	0.76	6.00E+08	6.00E+08	6.00E+08	3.97E+07
Petunidin 3-O-glucoside	12.92	479.118	[M]+	317	$C_{22}H_{23}O_{12}$+	1	0.884	1.00E+09	1.00E+09	1.00E+09	5.57E+07
Petunidin 3-O-arabinoside	12.36	449.1075	[M]+	317	$C_{21}H_{21}O_{11}$+	1	0.496	3.00E+08	3.00E+08	3.00E+08	2.46E+07
Malvidin 3-O-glucoside	13.71	493.1336	[M]+	331	$C_{23}H_{25}O_{12}$+	1	0.893	2.00E+09	2.00E+09	2.00E+09	1.12E+08
Peonidin 3-O-glucoside	13.73	463.1234	[M]+	301	$C_{22}H_{23}O_{11}$+	2	0.23	2.00E+08	2.00E+08	3.00E+08	1.44E+07
Malvidin 3-O-arabinoside	14.32	463.1232	[M]+	331	$C_{22}H_{23}O_{11}$+	2	0.731	6.00E+08	6.00E+08	5.00E+08	3.36E+07
Tetra-hydroxyflavone	14.49	319.0812	[M+H]+	-	$C_{16}H_{14}O_7$	3	0.333	6.53E+05	6.13E+05	6.92E+05	7.27E+04
Unknown (C)	14.87	511.1442	[M+H]+	349; 223; 493	$C_{23}H_{26}O_{13}$	3	0.901	2.00E+08	2.00E+08	2.00E+08	1.02E+07
Myricetin 3-O-glucoside	14.99	481.0974	[M+H]+	319	$C_{21}H_{20}O_{13}$	1	0.797	2.00E+08	2.00E+08	2.00E+08	1.63E+07
Myricetin fragment 1	14.99	319.0448	[M+H]+	-	$C_{15}H_{10}O_8$	3	0.569	1.31E+07	1.00E+07	1.00E+07	1.03E+06
Methyl-epicatechin glucuronide	15.26	481.1337	[M+H]+	-	$C_{22}H_{24}O_{12}$	3	0.33	7.51E+07	8.00E+07	7.00E+07	7.58E+06
Unknown (D)	15.8	331.154	[M+H]+	331; 287; 151; 189	$C_{19}H_{22}O_5$	4	0.874	9.28E+07	9.00E+07	9.00E+07	7.73E+06
Quercetin 3-O-rutinoside	15.91	611.1605	[M+H]+	303; 465	$C_{27}H_{30}O_{16}$	1	0.696	1.00E+08	1.00E+08	1.00E+08	7.91E+06
Myricetin fragment 2	15.97	319.0448	[M+H]+	-	$C_{15}H_{10}O_8$	3	0.989	1.30E+07	1.00E+07	1.00E+07	8.20E+05
Quercetin 3-O-glucoside	16.26	465.1024	[M+H]+	303	$C_{21}H_{20}O_{12}$	1	0.931	6.00E+08	6.00E+08	6.00E+08	3.96E+07

Vanoleic acid isomer A	16.42	771.1041	[M+H]+	305; 279; 261; 431; 449; 233; 413; 601; 619	$C_{34}H_{26}O_{21}$	3	0.464	1.25E+07	1.00E+07	1.00E+07	6.13E+05
Unknown (E)	16.78	538.228	[M+H]+	235; 341; 175; 323; 205	$C_{26}H_{35}NO_{11}$	4	0.409	8.20E+06	8.42E+06	7.98E+06	4.68E+05
Unknown (F)	16.78	341.1384	[M+H]+	323; 271; 291; 199; 177; 137	$C_{20}H_{20}O_{5}$	4	0.562	1.57E+07	2.00E+07	2.00E+07	9.22E+05
Vanoleic acid isomer B	16.81	771.1042	[M+H]+	-	$C_{34}H_{26}O_{21}$	3	0.993	4.09E+06	4.09E+06	4.09E+06	3.35E+05
Quercetin 3-O-arabinoside	17.38	435.092	[M+H]+	303	$C_{20}H_{18}O_{11}$	2	0.853	2.00E+08	2.00E+08	2.00E+08	1.52E+07
Kaempferol 3-O-glucoside	17.59	449.1076	[M+H]+	-	$C_{21}H_{20}O_{11}$	2	0.488	1.00E+08	1.00E+08	1.00E+08	9.33E+06
Syringetin 3-O-glucoside	17.69	509.1285	[M+H]+	347	$C_{23}H_{24}O_{13}$	3	0.982	1.00E+08	1.00E+08	1.00E+08	9.23E+06
Unknown (H)	17.85	957.3979	[M+H]+	-	$C_{45}H_{64}O_{22}$	4	0.739	1.03E+06	1.06E+06	9.99E+05	1.70E+05
Unknown (G)	17.92	839.3347	[M+H]+	-	$C_{40}H_{54}O_{19}$	4	0.988	3.06E+06	3.06E+06	3.07E+06	4.94E+05
Curcubitacin H isomer	17.98	535.3262	[M+H]+	517; 487; 469; 499; 451; 287	$C_{30}H_{46}O_{8}$	3	0.76	8.85E+06	8.72E+06	8.98E+06	7.84E+05
Unknown (L)	18.03	1045.4135	[M+H]+	-	$C_{48}H_{68}O_{25}$	4	0.691	1.13E+06	1.12E+06	1.15E+06	7.50E+04
Unknown (I)	18.19	957.3978	[M+H]+	-	$C_{45}H_{64}O_{22}$	4	0.935	1.33E+06	1.34E+06	1.32E+06	1.88E+05
Unknown (O)	18.36	665.2819	[M+H]+	-	$C_{36}H_{42}NO_{11}$	4	0.9	3.86E+06	3.89E+06	3.83E+06	5.12E+05
Myricetin	18.54	319.0449	[M+H]+	273; 301; 245; 153; 165; 263	$C_{15}H_{10}O_{8}$	1	0.913	2.71E+07	3.00E+07	3.00E+07	2.74E+06
Unknown (J)	18.69	957.398	[M+H]+	-	$C_{48}H_{62}NO_{19}$	4	0.316	1.21E+06	1.31E+06	1.12E+06	1.69E+05
Unknown (M)	18.87	679.2972	[M+H]+	647; 522; 464; 490	$C_{34}H_{46}O_{14}$	4	0.844	3.48E+07	4.00E+07	3.00E+07	2.94E+06
Unknown (K)	19.21	957.3973	[M+H]+	-	$C_{45}H_{64}O_{22}$	4	0.221	1.26E+06	1.34E+06	1.17E+06	1.17E+05
Unknown (N)	19.23	679.2975	[M+H]+	-	$C_{34}H_{46}O_{14}$	4	0.813	1.20E+07	1.00E+07	1.00E+07	1.21E+06
Myrianthic acid derivative A	19.36	684.4317	[M+H]+	631; 469; 451; 666	$C_{33}H_{63}O_{14}$	3	0.837	7.41E+06	7.32E+06	7.49E+06	7.72E+05
Trihydroxyursenedioic acid isomer	19.36	519.3313	[M+H]+	-	$C_{30}H_{46}O_{7}$	3	0.542	2.43E+07	2.00E+07	2.00E+07	2.05E+06
Quercetin isomer	19.76	303.0498	[M+H]+	257; 285; 229; 149; 153	$C_{15}H_{10}O_{7}$	2	0.757	6.00E+08	6.00E+08	6.00E+08	5.53E+07
Unknown (P)	19.96	247.1328	[M+H]+	229; 187; 191; 201; 211	$C_{15}H_{18}O_{3}$	4	0.741	4.37E+07	4.00E+07	4.00E+07	4.07E+06

Dihydrocurcubitacin F isomer	20.08	521.347	[M+H]+	475; 503; 485; 441; 467; 423	$C_{30}H_{48}O_7$	3	0.713	1.98E+07	2.00E+07	2.00E+07	2.03E+06
Unknown (Q)	20.19	795.3445	[M+H]+	763; 601; 672	$C_{39}H_{54}O_{17}$	4	0.56	7.05E+06	7.18E+06	6.92E+06	4.07E+05
Myrianthic acid derivative B	20.2	684.4318	[M+H]+	631; 469; 505; 649; 667	$C_{33}H_{63}O_{14}$	3	0.961	5.17E+06	5.18E+06	5.16E+06	3.58E+05
Trachelosperogenin derivative A	20.62	698.4109	[M+H]+	501; 519; 483; 455; 437	$C_{33}H_{61}O_{15}$	4	0.761	6.69E+06	6.78E+06	6.60E+06	5.31E+05
Trachelosperogenin derivative B	21.09	698.4108	[M+H]+	-	$C_{33}H_{61}O_{15}$	4	0.666	8.20E+06	8.06E+06	8.35E+06	6.11E+05
Trachelosperogenin derivative C	21.37	698.4107	[M+H]+	-	$C_{33}H_{61}O_{15}$	4	0.754	2.78E+07	3.00E+07	3.00E+07	1.92E+06
Pinostillbene hexoside pentoside 1	21.77	537.1964	[M+H]+	405; 243; 375	$C_{26}H_{32}O_{12}$	3	0.601	9.10E+06	9.44E+06	8.77E+06	1.18E+06
Pinostillbene hexoside pentoside 2	22.12	537.1965	[M+H]+	-	$C_{26}H_{32}O_{12}$	3	0.63	3.00E+06	3.13E+06	2.87E+06	4.99E+05
Unknown (S)	22.93	374.2901	[M+H]+	275; 293; 213; 356; 311; 328; 173; 195	$C_{20}H_{39}NO_5$	4	0.36	1.39E+07	1.00E+07	1.00E+07	1.01E+06
Unknown (R)	23.18	506.2957	[M+H]+	177; 295; 259; 460	$C_{24}H_{43}NO_{10}$	4	0.9	2.02E+07	2.00E+07	2.00E+07	1.63E+06
Trihydroxyursenedioic acid isomer	23.65	519.3314	[M+H]+	473; 501; 471; 483; 465; 453	$C_{30}H_{46}O_7$	3	0.471	4.22E+07	4.00E+07	5.00E+07	1.04E+07
Kaempferol	23.72	287.055	[M+H]+	213; 241; 165; 153; 258; 231; 121	$C_{15}H_{10}O_6$	1	0.605	4.81E+07	5.00E+07	5.00E+07	4.46E+06
Fupenzic acid isomer A	24.39	485.3259	[M+H]+	449; 467; 187; 199; 405	$C_{30}H_{44}O_5$	3	0.499	7.13E+06	6.74E+06	7.52E+06	1.05E+06
Tetrahydroxy ursenoic acid isomer A	25.31	505.3521	[M+H]+	469; 487; 459; 451	$C_{30}H_{48}O_6$	3	0.867	2.99E+07	3.00E+07	3.00E+07	3.73E+06
Dioxooleanenoic acid isomer	25.33	469.331	[M+H]+	451; 405; 407; 433; 423	$C_{30}H_{44}O_4$	3	0.82	2.82E+07	3.00E+07	3.00E+07	3.03E+06
Fupenzic acid isomer B	25.66	485.326	[M+H]+	449; 437; 441; 467; 187; 201; 405	$C_{30}H_{44}O_5$	3	0.823	1.55E+07	2.00E+07	2.00E+07	2.34E+06
Hydroxyflavone	25.87	241.0859	[M+H]+	226; 131; 137; 223; 195; 163	$C_{15}H_{12}O_3$	2	0.543	1.28E+07	1.00E+07	1.00E+07	4.79E+06

Unknown (T)	25.95	619.2384	[M+H]+	-	$C_{31}H_{38}O_{13}$	4	0.795	4.54E+06	4.62E+06	4.47E+06	5.34E+05
Unknown (U)	26.27	619.2385	[M+H]+	-	$C_{31}H_{38}O_{13}$	4	0.774	2.04E+06	2.08E+06	1.99E+06	2.88E+05
Tetrahydroxy ursenoic acid isomer B	26.61	505.3521	[M+H]+	469; 487; 459; 451; 423	$C_{30}H_{48}O_6$	3	0.617	5.18E+07	5.00E+07	5.00E+07	5.14E+06
Unknown (V)	26.61	255.1591	[M+H]+	195	$C_{14}H_{22}O_4$	4	0.301	8.55E+06	8.34E+06	8.77E+06	3.68E+05
Fupenzic acid isomer C	26.89	485.3259	[M+H]+	437; 467; 439; 449; 421; 391	$C_{30}H_{44}O_5$	3	0.756	6.29E+06	6.12E+06	6.46E+06	1.03E+06
Dihydroxy dioxoursenoic acid isomer	27.21	501.3208	[M+H]+	453; 483; 465; 455; 437; 419; 447	$C_{30}H_{44}O_6$	3	0.708	3.64E+07	4.00E+07	4.00E+07	3.80E+06
Trihydroxyursenedioic acid isomer	27.27	519.3314	[M+H]+	501; 483; 473; 455; 437	$C_{30}H_{46}O_7$	3	0.213	3.37E+07	4.00E+07	3.00E+07	2.39E+06
Unknown (W)	27.42	359.1489	[M+H]+	323; 257; 341; 219	$C_{20}H_{22}O_6$	4	0.627	1.76E+07	2.00E+07	2.00E+07	1.78E+06
Coumaroyl tormentic acid	28.47	635.394	[M+H]+	453; 407; 435	$C_{39}H_{54}O_7$	3	0.215	2.81E+07	3.00E+07	3.00E+07	1.58E+06
Docosatrienol	28.64	338.3418	[M+NH$_4$]+	321; 303	$C_{22}H_{40}O$	3	0.06	1.00E+08	8.00E+07	1.00E+08	1.87E+07
Octadecatrienol	29.17	282.2791	[M+NH$_4$]+	265; 247	$C_{18}H_{32}O$	3	0.082	2.00E+09	2.00E+09	1.00E+09	6.39E+08
Ganoderol	29.49	441.3725	[M+H]+	207; 219; 189	$C_{30}H_{48}O_2$	3	0.21	1.00E+08	7.00E+07	2.00E+08	7.59E+07
Hexadecadienol	29.75	256.2635	[M+NH$_4$]+	88; 102; 116; 130; 144; 158; 172; 186; 200; 214	$C_{16}H_{30}O$	3	0.107	2.00E+08	2.00E+08	1.00E+08	6.35E+07
Farnesyl methyl ether	30.71	254.2479	[M+NH$_4$]+	237; 219	$C_{16}H_{28}O$	3	0.299	2.76E+07	4.00E+07	2.00E+07	1.61E+07
Farnesyl acetone	30.92	280.2635	[M+NH$_4$]+	263; 245	$C_{18}H_{30}O$	3	0.193	1.55E+07	2.00E+07	9.00E+06	8.13E+06
Tetradecadienol	30.92	228.2322	[M+NH$_4$]+	88; 102; 116; 130; 144; 158; 172; 186; 193; 210	$C_{14}H_{26}O$	3	0.387	1.07E+07	1.00E+07	8.00E+06	5.10E+06
Unknown (X)	30.95	593.2755	[M+H]+	533	$C_{34}H_{40}O_9$	4	0.118	8.44E+06	1.00E+07	5.00E+06	2.96E+06
Unknown (Z)	31.8	182.9851	[M+H]+	160; 142	-	4	0.169	5.35E+07	6.00E+07	5.00E+07	2.56E+06

Supplementary Table S5 - List of tentatively annotated metabolites present in the chemical analysis in ESI negative mode of the different non-hydrolysed diets. Analysis of variance results comparing berry diets with either high salt or low salt composition include significance level (F pr.), grand mean, high salt mean, low salt mean and standard error of means (SEM) for each metabolite. Identification level corresponds to the levels of confidence on the annotation of the metabolite: 1- Annotation based on two or more orthogonal properties with an authentic chemical standard analysed under identical analytical conditions; 2 - based upon physicochemical properties and/or spectral similarity with public commercial spectral libraries, without reference to authentic chemical standards; 3 - based upon characteristic physicochemical properties of a chemical class of compounds, or by spectral similarity to know compounds of a chemical class; 4 - unidentified and unclassified, these metabolites can still be differentiated and quantified based upon spectral data.

Variate	Retention Time	m/z	adduct	ms2	Molecular Formula	Identification level	F pr.	Grand mean	High salt mean	Low salt mean	SEM
Galloyl glucose isomer A	3.72	331.0673	[M-H]-	169; 271; 211; 125; 193; 241; 313	$C_{13}H_{16}O_{10}$	2	0.85	4.50E+07	4.50E+07	4.60E+07	5.96E+06
Gallic acid	4.67	169.0152	[M-H]-	125	$C_7H_6O_5$	1	0.738	2.30E+07	2.40E+07	2.30E+07	2.62E+06
Galloyl glucose isomer B	4.88	331.0685	[M-H]-	169; 271; 125	$C_{13}H_{16}O_{10}$	2	0.33	2.20E+07	2.20E+07	2.10E+07	1.07E+06
Galloyl-quinic acid	6.36	343.0685	[M-H]-	191; 169	$C_{16}H_{16}O_{10}$	3	0.754	3.20E+08	3.20E+08	3.20E+08	2.30E+07
Galloyl glucose isomer C	7.01	331.0676	[M-H]-	169; 313; 125	$C_{13}H_{16}O_{10}$	2	0.369	5.80E+06	1.10E+07	2.30E+05	1.10E+07
Protocatechuic acid	8.63	153.0198	[M-H]-	-	$C_7H_6O_4$	1	0.832	6.90E+06	7.00E+06	6.80E+06	6.37E+05
Galloyl glucose isomer D	8.68	331.0677	[M-H]-	169; 125	$C_{13}H_{16}O_{10}$	2	0.696	4.20E+07	4.30E+07	4.10E+07	5.02E+06
Galloyl shikimate isomer A	9.09	325.0572	[M-H]-	169; 125	$C_{14}H_{14}O_9$	2	0.88	2.50E+07	2.50E+07	2.50E+07	3.12E+06
Galloyl shikimate isomer B	9.44	325.057	[M-H]-	-	$C_{14}H_{14}O_9$	2	0.829	4.60E+07	4.70E+07	4.50E+07	8.96E+06
Galloyl shikimate isomer C	9.72	325.0571	[M-H]-	-	$C_{14}H_{14}O_9$	2	0.642	6.30E+07	6.40E+07	6.20E+07	4.56E+06
Neochlorogenic acid	10.14	353.0886	[M-H]-	191	$C_{16}H_{18}O_9$	1	0.573	4.10E+07	4.10E+07	4.20E+07	2.77E+06
Unknown (A)	11.61	481.0999	[M-H]-	301; 355; 175; 329; 257; 319	$C_{21}H_{22}O_{13}$	4	0.891	2.50E+08	2.50E+08	2.50E+08	9.66E+06
Catechin	11.97	289.0724	[M-H]-	245; 205; 179; 203	$C_{15}H_{14}O_6$	1	0.79	1.10E+08	1.10E+08	1.10E+08	3.97E+06
Chlorogenic acid	12.21	353.0887	[M-H]-	191	$C_{16}H_{18}O_9$	1	0.781	4.80E+08	4.80E+08	4.80E+08	1.51E+07
Cyanidin 3-O-glucoside	12.64	447.0939	[M+-2H]-	401; 285; 269	$C_{21}H_{21}O_{11}^+$	1	0.294	1.80E+08	1.80E+08	1.80E+08	3.42E+06
Caffeic acid	12.94	179.0353	[M-H]-	-	$C_9H_8O_4$	1	0.282	5.00E+06	5.20E+06	4.70E+06	4.31E+05
Caffeoyl-quinate	13.07	353.0886	[M-H]-	-	$C_{16}H_{18}O_9$	2	0.526	8.60E+07	8.20E+07	9.10E+07	1.29E+07
Dihydromyricetin	13.71	319.0466	[M-H]-	-	$C_{15}H_{12}O_8$	1	0.394	6.80E+06	7.10E+06	6.60E+06	5.72E+05
Unknown (B)	14.12	366.1201	[M-H]-	186; 204; 142; 246	$C_{17}H_{21}NO_8$	4	0.452	1.30E+08	1.40E+08	1.30E+08	4.99E+06
Sanguiin H6	14.54	934.0721	[M-2H]2-	1567; 633; 301; 1235; 897; 1265; 915	$C_{82}H_{54}O_{52}$	2	0.054	6.30E+07	6.60E+07	6.00E+07	2.55E+06
Unknown (C)	14.75	627.2305	[M-H]-	581; 591; 609; 300	$C_{29}H_{40}O_{15}$	4	0.957	7.30E+07	7.30E+07	7.30E+07	3.97E+06
Unknown (D)	14.89	509.1311	[M-H]-	329; 463; 355; 347	$C_{23}H_{26}O_{13}$	4	0.658	1.40E+08	1.40E+08	1.30E+08	6.72E+06

Myricetin 3-O-glucoside	15.04	479.0839	[M-H]-	316, 317	$C_{21}H_{20}O_{13}$	1	0.678	1.70E+08	1.70E+08	1.70E+08	1.07E+07
Feruloyl glucose isomer A	15.19	355.1041	[M-H]-	193	$C_{16}H_{20}O_{9}$	2	0.657	5.30E+07	5.40E+07	5.20E+07	3.13E+06
p-Coumaric acid	15.22	163.0408	[M-H]-	-	$C_{9}H_{8}O_{3}$	1	0.268	2.50E+06	2.60E+06	2.40E+06	1.84E+05
Trigalloyl shikimate	15.26	629.0808	[M-H]-	477; 325	$C_{28}H_{22}O_{17}$	2	0.161	4.40E+07	4.50E+07	4.20E+07	1.75E+06
Tetragalloyl glucose	15.33	787.103	[M-H]-	617; 635; 465	$C_{34}H_{28}O_{22}$	2	0.129	1.60E+07	1.70E+07	1.60E+07	5.96E+05
Feruloyl glucose isomer B	15.82	355.1042	[M-H]-	-	$C_{16}H_{20}O_{9}$	2	0.642	8.20E+07	8.30E+07	8.00E+07	6.38E+06
Quercetin 3-O-rutinoside	15.95	609.1486	[M-H]-	301; 300; 343; 271	$C_{27}H_{30}O_{16}$	1	0.385	1.10E+08	1.10E+08	1.00E+08	5.43E+06
Ferulic acid	16.15	193.0514	[M-H]-	-	$C_{10}H_{10}O_{4}$	1	0.841	1.40E+06	1.40E+06	1.50E+06	3.15E+05
Quercetin 3-O-galactoside	16.31	463.09	[M-H]-	316; 317	$C_{21}H_{20}O_{12}$	1	0.34	3.00E+08	2.40E+08	3.70E+08	1.19E+08
Ellagic acid	16.43	301.0001	[M-H]-	257; 229; 179; 185; 151; 272; 284	$C_{14}H_{6}O_{8}$	1	0.605	2.50E+07	2.70E+07	2.40E+07	3.96E+06
Quercetin 3-O-arabinoside	17.4	433.0792	[M-H]-	301; 300	$C_{20}H_{18}O_{11}$	2	0.51	1.30E+08	1.40E+08	1.30E+08	6.33E+06
Kaempferol 3-O-glucoside	17.62	447.0946	[M-H]-	315; 284	$C_{21}H_{20}O_{11}$	2	0.939	1.00E+08	1.00E+08	1.00E+08	7.30E+06
Unknown (E)	18	579.3197	[M-H]-	-	$C_{31}H_{48}O_{10}$	4	0.701	6.60E+06	6.50E+06	6.70E+06	6.03E+05
Myricetin	18.55	317.0313	[M-H]-	179; 151; 192	$C_{15}H_{10}O_{8}$	1	0.371	3.40E+07	3.60E+07	3.30E+07	2.82E+06
Unknown (F)	18.99	451.1053	[M-H]-	341	$C_{24}H_{20}O_{9}$	4	0.815	2.50E+07	2.50E+07	2.60E+07	2.07E+06
Unknown (G)	19.41	711.399	[M+COOH]-	503; 665	$C_{37}H_{60}O_{13}$	4	0.315	6.10E+07	6.20E+07	5.90E+07	2.85E+06
Morin	19.79	301.0362	[M-H]-	229; 151; 125; 257; 273; 283	$C_{15}H_{10}O_{7}$	1	0.364	5.00E+08	4.80E+08	5.20E+08	4.38E+07
Eriodictyol	20.32	287.0568	[M-H]-	-	$C_{15}H_{12}O_{6}$	3	0.804	3.69E+05	3.40E+05	4.00E+05	2.15E+05
Unknown (H)	21.39	725.3766	[M-H]-	-	$C_{37}H_{58}O_{14}$	4	0.508	9.40E+07	9.50E+07	9.20E+07	5.25E+06
Unknown (I)	22.42	709.3815	[M-H]-	-	$C_{37}H_{58}O_{13}$	4	0.657	8.90E+06	9.10E+06	8.70E+06	8.33E+05
Trihydroxy octadecadienoic acid	22.94	327.2182	[M-H]-	171; 229; 291; 211; 309	$C_{18}H_{32}O_{5}$	3	0.166	6.30E+07	6.50E+07	6.00E+07	2.86E+06
Unknown (J)	23.2	533.2616	[M-COOH]-	487; 355; 311	$C_{24}H_{40}O_{10}$	4	0.518	3.60E+07	3.60E+07	3.50E+07	2.38E+06
Trihydroxyursenedioic acid isomer	23.69	563.3235	[M-COOH]-	517; 487	$C_{30}H_{46}O_{7}$	3	0.501	2.70E+07	2.50E+07	3.00E+07	6.62E+06
Kaempferol	23.73	285.041	[M-H]-	151; 229; 257; 265; 213; 169; 185	$C_{15}H_{10}O_{6}$	1	0.798	4.90E+07	4.80E+07	4.90E+07	3.53E+06
Trihydroxy octadecenoic acid	24.22	329.2338	[M-H]-	229; 211; 171; 311; 293	$C_{18}H_{34}O_{5}$	3	0.195	7.20E+07	7.50E+07	6.90E+07	3.76E+06
Hydroxyflavonol	26.62	253.1451	[M-H]-	209	$C_{14}H_{22}O_{4}$	4	0.936	4.65E+05	4.90E+05	4.40E+05	6.58E+05

Supplementary Table S6 - Characterization and quantification of anthocyanins in the berry mixture. Anthocyanins were analysed by UPLC-MS/MS. The analytical column was Acquity BEH UPLC (100 x 2.1 mm, 1.7 µm), the mobile phase was 10% acetic acid (eluent A) and acetonitrile (eluent B) [26]. The injection volume was 2.5 µL and the flow rate was 0.3 mL/min.

**Anthocyanins**	**Concentration (mg/kg) ± SEM**
Cyanidin glucoside	1053 ± 87.90
Cyanidin rutinoside	278 ± 7.44
Cyanidin arabinoside	42.8 ± 2.88
Cyanidin malonylglucoside	1.90 ± 0.17
Cyanidin acetylglucoside	0.92 ± 0.03
Delphinidin glucoside	915 ± 64.40
Delphinidin arabinoside	305 ± 18.40
Delphinidin acetylglucoside	4.73 ± 0.24
Pelargonidin glucoside	9.27 ± 2.08
Pelargonidin rutinoside	3.52 ± 0.18
Pelargonidin malonylglucoside	1.67 ± 0.27
Pelargonidin arabinoside	0.29 ± 0.01
Pelargonidin acetylglucoside	0.15 ± 0.00
Petunidin glucoside	913 ± 68.10
Petunidin arabinoside	140 ± 9.27
Petunidin acetylglucoside	5.19 ± 0.11
Peonidin glucoside	67.3 ± 4.09
Peonidin arabinoside	5.26 ± 0.51
Peonidin acetylglucoside	0.66 ± 0.02
Malvidin glucoside	859 ± 63.10
Malvidin arabinoside	126 ± 7.58
Malvidin acetylglucoside	2.04 ± 0.21
Total anthocyanins	4734
2g of lyophilized berries mixture*	9.47 mg

*2 g of lyophilized berries added to 50g of diet.

Supplementary Table S7 - Characterization and quantification of main aglycones in the berry mixture, after enzymatic hydrolysis using HPLC-DAD relative to authentic standards as previously described [60].

	**Concentration (mg/kg)**
**Phenolic acids**	**6725**
Gallic acid	4259
Protocatechuic acid	167
3-O-Methylgallic acid	129
Vanillic acid	29
Caffeic acid	1404
Syringic acid	132
Ellagic acid	248
Ferulic acid	69
Phloroglucinaldehyde	288
**Flavanols**	**298**
(+)-Catechin	176
(-)-Epicatechin	122
**Flavonols**	**335**
Myricetin	156
Quercetin	155
Kaempferol	24

Supplementary Table S8 - List of tentatively annotated metabolites present in the chemical analysis in ESI positive mode of the different hydrolysed diets. Analysis of variance results comparing berry diets with either high salt or low salt composition include significance level (F pr.), grand mean, high salt mean, low salt mean and standard error of means (SEM for each metabolite. Identification level corresponds to the levels of confidence on the annotation of the metabolite: 1- Annotation based on two or more orthogonal properties with an authentic chemical standard analysed under identical analytical conditions; 2 - based upon physicochemical properties and/or spectral similarity with public commercial spectral libraries, without reference to authentic chemical standards; 3 - based upon characteristic physicochemical properties of a chemical class of compounds, or by spectral similarity to know compounds of a chemical class; 4 - unidentified and unclassified, these metabolites can still be differentiated and quantified based upon spectral data.

Compound	Retention Time	m/z	adduct	ms2	Molecular Formula	Identification level	F pr.	Grand mean	High salt mean	Low salt mean	SEM
Epigallocatechin isomer 1	8.77	307.0813	[M+H]+	-	$C_{15}H_{14}O_7$	3	0.27	1.12E-02	1.41E-02	8.20E-03	4.63E-03
Epigallocatechin isomer 2	9.16	307.0813	[M+H]+	139; 151; 289	$C_{15}H_{14}O_7$	3	0.453	2.42E-02	2.80E-02	2.04E-02	9.14E-03
Epicatechin dimer 1	9.83	595.1443	[M+H]+	-	$C_{30}H_{26}O_{13}$	3	0.745	1.21E-02	1.41E-02	1.01E-02	1.14E-02
Methyl gallate isomer 1	10.19	185.0445	[M+H]+	-	$C_8H_8O_5$	3	0.195	1.30E-02	1.58E-02	1.02E-02	3.56E-03
Epicatechin dimer 2	10.22	595.1448	[M+H]+		$C_{30}H_{26}O_{13}$	3	0.752	7.90E-04	7.30E-04	8.50E-04	3.59E-04
Epicatechin dimer 3	11.18	579.1494	[M+H]+	427; 409; 291; 247; 301	$C_{30}H_{26}O_{12}$	3	0.919	3.27E-02	3.32E-02	3.23E-02	8.23E-03
Methyl gallate 2	11.39	185.0445	[M+H]+	153; 171; 141	$C_8H_8O_5$	3	0.913	4.90E-02	4.60E-02	5.10E-02	4.21E-02
Epicatechin dimer 4	11.51	579.1494	[M+H]+	-	$C_{30}H_{26}O_{12}$	3	0.555	3.88E-02	4.30E-02	3.47E-02	1.28E-02
Catechin isomer 1	11.78	291.0863	[M+H]+	139; 123; 165; 151; 273	$C_{15}H_{14}O_6$	1	0.235	1.44E-01	1.26E-01	1.62E-01	2.57E-02
Catechin isomer 2	11.89	291.0863	[M+H]+	-	$C_{15}H_{14}O_6$	1	0.301	2.49E-01	2.80E-01	2.18E-01	5.21E-02
Chlorogenic acid isomer 1	11.98	355.1024	[M+H]+	163; 193	$C_{16}H_{18}O_9$	1	0.637	1.08E-02	1.35E-02	8.10E-03	1.07E-02
Procyanidin C isomer 1	12.05	867.2134	[M+H]+		$C_{45}H_{38}O_{18}$	3	0.878	9.20E-03	9.60E-03	8.90E-03	4.24E-03
Chlorogenic acid isomer 2	12.18	355.1024	[M+H]+	-	$C_{16}H_{18}O_9$	1	0.634	1.54E-02	1.92E-02	1.16E-02	1.46E-02
Procyanidin B1 isomer 1	12.43	579.1493	[M+H]+	-	$C_{30}H_{26}O_{12}$	3	0.668	1.10E-02	1.14E-02	1.05E-02	1.94E-03
Delphinidin isomer 1	12.68	303.0499	M+	177; 153	$C_{15}H_{11}O_7$	2	0.788	5.90E-02	5.30E-02	6.50E-02	3.98E-02
Dihydromyricetin isomer	12.74	321.0605	[M+H]+	153; 303	$C_{15}H_{12}O_8$	3	0.975	1.13E-01	1.14E-01	1.11E-01	7.88E-02
Caffeic Acid isomer 1	12.87	181.0495	[M+H]+	163	$C_9H_8O_4$	1	0.79	5.23E-01	5.35E-01	5.10E-01	8.64E-02
Procyanidin B	12.94	593.1148	[M+H]+	-	$C_{30}H_{24}O_{13}$	2	0.562	5.40E-03	6.40E-03	4.50E-03	3.11E-03
Unknown 1	12.98	403.1023	[M+H]+	251; 263; 385	$C_{20}H_{18}O_9$	4	0.764	4.80E-02	4.20E-02	5.40E-02	3.70E-02
Syringic acid isomer 1	13.26	199.0601	[M+H]+	155; 173; 140	$C_9H_{10}O_5$	3	0.163	4.02E-02	4.71E-02	3.33E-02	8.07E-03
Dihydroxy Flavone Hexoside	13.31	471.1284	[M+H]+	-	$C_{24}H_{22}O_{10}$	3	0.994	3.40E-03	3.40E-03	3.40E-03	2.44E-03
Procyanidin B1 isomer 2	13.31	579.1498	[M+H]+	453; 409; 435; 427; 561; 301	$C_{30}H_{26}O_{12}$	3	0.307	1.16E-02	1.09E-02	1.23E-02	1.17E-03
Epicatechin isomer 1	13.34	291.0864	[M+H]+	139; 123; 165; 273; 151	$C_{15}H_{14}O_6$	2	0.636	5.95E-02	6.26E-02	5.64E-02	1.20E-02
Trimethoxyphenylacetic_acid	13.38	227.0914	[M+H]+	155; 209	$C_{11}H_{14}O_5$	3	0.339	1.32E-02	1.51E-02	1.13E-02	3.51E-03

Dihydromyricetin	13.68	321.0605	[M+H]+	303; 275; 153; 195	$C_{15}H_{12}O_8$	1	0.45	2.05E-02	2.09E-02	2.01E-02	9.31E-04
Malvidin-glucoside	13.74	493.1338	M+	331	$C_{23}H_{25}O_{12}^+$	1	0.547	3.60E-01	5.10E-01	2.10E-01	4.43E-01
Procyanidin C isomer 2	13.74	867.2133	[M+H]+	-	$C_{45}H_{38}O_{18}$	3	0.805	7.72E-03	7.94E-03	7.51E-03	1.65E-03
Protocatechuic acid isomer	14.18	155.0339	[M+H]+	127	$C_7H_6O_4$	2	0.472	2.15E-01	2.58E-01	1.71E-01	1.10E-01
Procyanidin B1 isomer 3	14.27	579.1495	[M+H]+	409; 427; 453; 561; 291	$C_{30}H_{26}O_{12}$	3	0.739	2.22E-02	2.17E-02	2.26E-02	2.52E-03
Cyanidin	14.48	287.0551	M+	177; 137	$C_{15}H_{11}O_6^+$	3	0.71	1.29E-01	1.12E-01	1.45E-01	8.17E-02
Taxifolin isomer	14.49	305.0656	[M+H]+	137; 287	$C_{15}H_{12}O_7$	3	0.786	1.48E-01	1.34E-01	1.62E-01	9.64E-02
Delphinidin	14.7	303.05	M+	285; 193; 267	$C_{15}H_{11}O_7^+$	3	0.376	3.74E-02	4.26E-02	3.22E-02	1.04E-02
Unknown 2	14.76	332.2068	[M+NH4]+	287	$C_{16}H_{26}O_6$	4	0.744	1.50E-02	1.60E-02	1.40E-02	5.62E-03
Methoxy-Taxifolin	15.14	335.0762	[M+H]+	167; 317; 275	$C_{16}H_{14}O_8$	3	0.816	1.08E-01	9.90E-02	1.16E-01	6.89E-02
Petunidin	15.14	317.0656	M+	177; 167	$C_{16}H_{13}O_7^+$	1	0.697	7.90E-02	6.90E-02	8.90E-02	4.87E-02
Coumaric acid	15.16	165.0546	[M+H]+	147	$C_9H_8O_3$	2	0.989	1.01E-01	1.01E-01	1.01E-01	1.24E-02
Cyanidin isomer	15.53	287.0551	M+	-	$C_{15}H_{11}O_6^+$	1	0.418	3.90E-02	6.80E-02	1.10E-02	6.41E-02
Feruloyl-glucoside	15.74	357.1181	[M+H]+	195; 163	$C_{16}H_{20}O_9$	2	0.471	2.27E-02	2.94E-02	1.59E-02	1.70E-02
Quercetin-glucoside isomer 1	15.82	465.1026	[M+H]+	303	$C_{21}H_{20}O_{12}$	2	0.245	3.67E-02	4.79E-02	2.54E-02	1.66E-02
Epicatechin dimer isomer 5	15.85	579.1498	[M+H]+	-	$C_{30}H_{26}O_{12}$	3	0.959	2.95E-03	2.94E-03	2.97E-03	4.99E-04
Unknown 3	15.85	317.1385	[M+H]+	299; 151; 137; 175; 271	$C_{18}H_{20}O_5$	4	0.605	8.90E-03	1.24E-02	5.40E-03	1.24E-02
Methyl Epicatechin-Epicatechin	15.87	593.1654	[M+H]+	-	$C_{31}H_{28}O_{12}$	3	0.416	1.46E-03	1.09E-03	1.82E-03	8.03E-04
Methoxy-indoleacetate	15.87	206.0812	[M+H]+	-	$C_{11}H_{11}NO_3$	4	0.189	7.67E-03	8.56E-03	6.78E-03	1.12E-03
Unknown 4	15.9	249.1121	[M+H]+	187; 159; 189; 231; 221	$C_{14}H_{16}O_4$	4	0.764	2.41E-02	2.67E-02	2.14E-02	1.64E-02
Unknown 5	15.9	466.2433	[M+H]+	249; 267	$C_{24}H_{35}NO_8$	4	0.779	3.29E-02	3.37E-02	3.20E-02	5.75E-03
(2E)-3-(4-Hydroxy-3-methoxyphenyl)-propanoic acid	15.96	193.0495	[M+H]+	133; 165; 178; 149	$C_{10}H_8O_4$	2	0.552	1.70E-02	1.80E-02	1.60E-02	3.10E-03
Quercetin 3-O-Galactoside or Hyperoside	16	465.1026	[M+H]+	303; 319	$C_{21}H_{20}O_{12}$	1	0.396	1.02E-01	1.18E-01	8.60E-02	3.41E-02
Ferulic acid	16.08	195.0651	[M+H]+	177	$C_{10}H_{10}O_4$	1	0.633	1.61E-01	1.66E-01	1.55E-01	2.24E-02

Supplementary Table S9 - List of tentatively annotated metabolites present in the chemical analysis in ESI negative mode of the different hydrolysed diets. Analysis of variance results comparing berry diets with either high salt or low salt composition include significance level (F pr.), grand mean, high salt mean, low salt mean and standard error of means (SEM) for each metabolite. Identification level corresponds to the levels of confidence on the annotation of the metabolite: 1- Annotation based on two or more orthogonal properties with an authentic chemical standard analysed under identical analytical conditions; 2 - based upon physicochemical properties and/or spectral similarity with public commercial spectral libraries, without reference to authentic chemical standards; 3 - based upon characteristic physicochemical properties of a chemical class of compounds, or by spectral similarity to know compounds of a chemical class; 4 - unidentified and unclassified, these metabolites can still be differentiated and quantified based upon spectral data.

Compound	Retention Time	m/z	adduct	ms2	Molecular Formula	Identification level	F pr.	Grand mean	High salt mean	Low salt mean	SEM.
Gallic acid isomer 1	2.75	169.0146	[M-H]-	125	$C_7H_6O_5$	2	0.032	2.00E+08	3.00E+08	2.00E+08	2.55E+07
Gallic acid	4.69	169.0151	[M-H]-	125	$C_7H_6O_5$	1	0.121	2.00E+08	2.00E+08	2.00E+08	1.11E+07
Protocatechuic acid isomer 1	8.18	153.0201	[M-H]-	109	$C_7H_6O_4$	1	0.411	2.38E+07	3.00E+07	2.00E+07	7.85E+06
Protocatechuic acid isomer 2	8.6	153.0201	[M-H]-	-	$C_7H_6O_4$	1	0.465	3.38E+07	4.00E+07	3.00E+07	1.34E+07
Epigallocatechin	9.18	305.0677	[M-H]-	179; 221; 261; 287; 165; 125	$C_{15}H_{14}O_7$	1	0.398	6.62E+06	6.85E+06	6.40E+06	4.75E+05
Epicatechin-Epicatechin	9.86	593.1315	[M-H]-	-	$C_{30}H_{26}O_{13}$	3	0.746	1.44E+06	1.55E+06	1.34E+06	6.11E+05
Methyl-gallate isomer 1	10.24	183.0307	[M-H]-	168; 139; 124	$C_8H_8O_5$	3	0.247	1.51E+07	2.00E+07	1.00E+07	4.98E+06
Hydroxy-benzoic acid	10.55	137.0251	[M-H]-	-	$C_7H_6O_3$	3	0.907	3.91E+06	3.84E+06	3.98E+06	1.18E+06
Methyl-gallate isomer 2	11.41	183.0308	[M-H]-	168; 139; 124	$C_8H_8O_5$	3	0.967	1.00E+08	1.00E+08	1.00E+08	1.34E+08
Procyanidin B	11.56	577.1371	[M-H]-	425; 407; 451; 397; 289; 559	$C_{30}H_{26}O_{12}$	3	0.831	7.49E+06	7.63E+06	7.35E+06	1.24E+06
Catechin isomer 1	11.77	289.0729	[M-H]-	245; 205; 179	$C_{15}H_{14}O_6$	1	0.787	4.17E+07	4.00E+07	4.00E+07	1.86E+07
Catechin isomer 2	11.93	289.073	[M-H]-	245;205; 179	$C_{15}H_{14}O_6$	1	0.832	5.08E+07	5.00E+07	5.00E+07	5.31E+06
Hydroxyphenyloxoacetic acid	12.06	165.0202	[M-H]-	-	$C_8H_6O_4$	3	0.879	8.71E+06	8.21E+06	9.20E+06	6.08E+06
Trihydroxycoumarin isomer 1	12.4	193.0151	[M-H]-	-	$C_9H_6O_5$	3	0.38	3.70E+06	3.26E+06	4.14E+06	8.90E+05
Dihydromyricetin isomer	12.76	319.0473	[M-H]-	193; 167; 301; 275	$C_{15}H_{12}O_8$	2	0.824	2.00E+08	1.00E+08	2.00E+08	1.02E+08
Caffeic acid	12.91	179.0358	[M-H]-	135	$C_9H_8O_4$	2	0.977	6.00E+08	6.00E+08	6.00E+08	1.32E+08
Epicatechin	13.37	289.0729	[M-H]-	245; 205; 179	$C_{15}H_{14}O_6$	2	0.952	1.21E+07	1.00E+07	1.00E+07	5.43E+06

Dihydromyricetin	13.69	319.0471	[M-H]-	193; 301	$C_{15}H_{12}O_8$	1	0.938	9.44E+06	9.33E+06	9.54E+06	2.60E+06
Protocatechuic acid isomer 3	14.21	153.0201	[M-H]-	109; 125; 107; 83	$C_7H_6O_4$	2	0.487	1.00E+08	2.00E+08	9.00E+07	8.97E+07
Unknown 1	14.21	225.1142	[M-H]-	181; 165; 147; 135; 207	$C_{12}H_{18}O_4$	4	0.889	4.25E+07	4.00E+07	4.00E+07	8.83E+06
Taxifolin isomer	14.48	303.0523	[M-H]-	167; 193; 259; 285	$C_{15}H_{12}O7$	2	0.879	2.00E+08	2.00E+08	2.00E+08	1.35E+08
Dihydroxyphenylacetic acid isomer 1	14.76	167.0357	[M-H]-	152; 108	$C_8H_8O_4$	3	0.886	2.09E+07	2.00E+07	2.00E+07	1.30E+07
6,6'-Dihydroxy-5,5'-dimethoxy-3,3'-biphenyldicarboxylic acid	15.16	333.0627	[M-H]-	167; 193; 289; 305; 207; 315	$C_{16}H_{14}O_8$	3	0.849	1.00E+08	1.00E+08	1.00E+08	9.24E+07
Cyanidin isomer	15.54	285.0409	[M+-2H]-	-	$C_{15}H_{11}O_6^{+}$	4	0.392	1.32E+07	2.00E+07	2.00E+06	2.40E+07
Unknown 2	15.56	363.1464	[M-H]-	-	$C_{19}H_{24}O_7$	4	0.785	3.69E+06	3.53E+06	3.85E+06	1.11E+06
Myricetin isomer 1	15.77	317.031	[M-H]-	-	$C_{15}H_{10}O_8$	4	0.535	1.68E+06	1.98E+06	1.39E+06	8.72E+05
Unknown 3	15.78	567.2103	[M-H]-	-	$C_{27}H_{36}O_{13}$	4	0.705	1.38E+07	2.00E+07	1.00E+07	1.32E+07
Unknown 4	15.88	204.0675	[M-H]-	-	$C_{11}H_{11}NO_3$	4	0.637	5.26E+06	5.87E+06	4.66E+06	2.39E+06
Hyperoside	16.04	463.09	[M-H]-	300; 343; 373	$C_{21}H_{20}O_{12}$	2	0.549	2.98E+07	3.00E+07	3.00E+07	5.14E+06
Ferulic acid	16.12	193.0515	[M-H]-	149; 178; 134	$C_{10}H_{10}O_4$	2	0.927	1.23E+07	1.00E+07	1.00E+07	3.44E+06
Ellagic acid	16.39	301.0001	[M-H]-	257; 229; 185; 284	$C_{14}H_6O_8$	2	0.885	8.32E+07	8.00E+07	8.00E+07	8.87E+06
Dihydroxyphenylacetic acid isomer 2	16.42	167.0357	[M-H]-	-	$C_8H_8O_4$	3	0.939	3.59E+06	3.53E+06	3.65E+06	1.40E+06
Syringetin isomer 1	16.81	345.0629	[M-H]-	330	$C_{17}H_{14}O_8$	2	0.031	1.36E+07	2.00E+07	1.00E+07	2.14E+06
Gallic acid isomer 2	16.91	169.015	[M-H]-	125; 151	$C_7H_6O_5$	2	0.223	6.75E+06	6.32E+06	7.17E+06	5.90E+05
Unknown 5	16.92	363.0735	[M-H]-	165; 197; 183; 319; 137	$C_{17}H_{16}O_9$	4	0.397	1.35E+07	2.00E+07	1.00E+07	4.40E+06
Myricetin isomer 2	17.07	317.0316	[M-H]-	-	$C_{15}H_{10}O_8$	3	0.912	2.74E+07	3.00E+07	3.00E+07	1.66E+07
Unknown 6	17.16	451.1992	[M-H]-	341; 299	$C_{23}H_{32}O_9$	4	0.967	9.93E+06	9.85E+06	1.00E+07	3.20E+06
Asterric acid	17.43	347.0787	[M-H]-	261; 303; 165; 193	$C_{17}H_{16}O_8$	3	0.862	2.00E+08	2.00E+08	2.00E+08	1.37E+08
Quercitrin	17.61	447.0951	[M-H]-	301	$C_{21}H_{20}O_{11}$	2	0.779	4.29E+07	4.00E+07	4.00E+07	1.01E+07
Syringetin 3-O-glucoside	17.69	507.1165	[M-H]-	344; 387; 479	$C_{23}H_{24}O_{13}$	3	0.351	4.00E+07	4.00E+07	4.00E+07	4.40E+06
Unknown 7	17.73	571.2207	[M-H]-	523; 345; 357; 375	$C_{30}H_{36}O_{11}$	4	0.912	4.64E+06	4.57E+06	4.71E+06	1.21E+06
Gentiin	17.9	257.0468	[M-H]-	-	$C_{14}H_{10}O_5$	3	0.515	1.63E+06	1.42E+06	1.83E+06	5.75E+05

Unknown 8	17.97	579.32	[M-H]-	533; 417; 503; 561; 463	$C_{31}H_{48}O_{10}$	4	0.993	4.69E+06	4.69E+06	4.68E+06	8.52E+05
Myricetin isomer 2	18.11	317.0315	[M-H]-	299, 195, 271	$C_{15}H_{10}O_8$	2	0.624	2.01E+07	2.00E+07	2.00E+07	4.87E+06
Eriodictyol isomer	18.33	287.0573	[M-H]-	259, 243, 269	$C_{15}H_{12}O_6$	2	0.729	1.05E+07	1.00E+07	1.00E+07	3.20E+06
Ferulic acid isomer	18.46	193.0516	[M-H]-	161, 134, 178, 149	$C_{10}H_{10}O_4$	2	0.818	2.00E+08	2.00E+08	2.00E+08	1.74E+08
Myricetin	18.53	317.0314	[M-H]-	179, 151, 192	$C_{15}H_{10}O_8$	1	0.651	2.00E+08	1.00E+08	2.00E+08	3.55E+07
Unknown 9	18.87	677.285	[M-H]-	645, 617, 659, 585	$C_{27}H_{50}O_{19}$	4	0.606	2.96E+06	3.16E+06	2.76E+06	7.28E+05
